# TiO_2_ particles induce CHIT1 and YKL-40 in human macrophages: implications for implant-associated inflammation

**DOI:** 10.3389/fimmu.2026.1964504

**Published:** 2026-10-28

**Authors:** Tatyana N. Sevastyanova, Lina S. Silva-Bermudez, Zhen Jiao, Christina Schmuttermaier, Leonie Schumacher, Udo Obertacke, Sascha Gravius, Andreas Schilder, Harald Klüter, Julia Kzhyshkowska

**Affiliations:** 1 Institute of Transfusion Medicine and Immunology, Medical Faculty Mannheim, Heidelberg University, Mannheim, Germany; 2 Department of Orthopedics and Trauma Surgery, Universitätsklinikum Heidelberg – UK Mannheim GmbH, Medical Faculty Mannheim, Heidelberg University, Mannheim, Germany; 3 German Red Cross Blood Service Baden-Württemberg – Hessen, Mannheim, Germany

**Keywords:** biomaterials, chitinase-3-like protein 1 (CHI3L1/YKL-40), chitotriosidase (CHIT1), implant-related inflammation, macrophages, TiO_2_ particles

## Abstract

Titanium is widely used for orthopedic and dental implants because of its biocompatibility and mechanical properties. However, corrosion and mechanical wear can result in the release of titanium-containing particulate debris, which may contribute to chronic peri-implant inflammation and implant failure. Macrophages are primary innate immune cells that recognize and respond to implant-derived particles, yet the effects of TiO_2_ particles on the macrophage-derived chitinase family members chitotriosidase (CHIT1) and YKL-40 (CHI3L1) remain poorly understood. Here, we investigated the effects of TiO_2_ particles derived from a 15-nm rutile nanoparticulate starting material that exhibited substantial and preparation-dependent agglomeration during laser diffraction analysis on primary human M0, M1, and M2 macrophages. Metabolic activity, assessed using the Alamar Blue assay, was maintained or increased following exposure to 25, 50, 100, and 200 ppm TiO_2_, with a statistically significant increase observed at 100 ppm in M0 macrophages, while no significant changes were detected in M1 or M2 macrophages. Transcriptomic analysis identified CHIT1 and CHI3L1 as the two most upregulated members of the Glyco_18 protein family. RT-qPCR confirmed significant induction of CHIT1 following TiO_2_ exposure in M0, M1, and M2 macrophages, while CHI3L1 expression was significantly increased in M0 and M2 macrophages. Increased gene expression was accompanied by a concentration-dependent increase in YKL-40 secretion, with statistically significant increases observed from 100 ppm TiO_2_ onwards. At 100 ppm, extracellular CHIT1 protein levels increased significantly in M0 and M1 macrophages, despite no corresponding significant increase in CHIT1 enzymatic activity. Immunofluorescence analysis revealed altered intracellular localization of CHIT1 following TiO_2_ exposure, with accumulation in the trans-Golgi network (TGN) of M1 macrophages and disruption of TGN organization in M0 and M2 macrophages. Furthermore, CHIT1 and YKL-40 were detected in CD68-positive macrophages within peri-implant tissues obtained from patients undergoing removal of titanium fracture fixation implants, supporting the clinical relevance of the *in vitro* findings. Collectively, these findings identify CHIT1 and YKL-40 as macrophage-associated proteins responsive to TiO_2_ particle exposure and support their further investigation in biomaterial-associated inflammation.

## 1 Introduction

During metal degradation, also referred to as corrosion, metal microparticles, nanoparticles, and ions are continuously released from implant surfaces as a consequence of electrochemical interactions with the surrounding biological environment, and in particular with immune microenvironment (1–4). Despite the excellent mechanical properties, corrosion resistance, and low magnetic susceptibility of titanium and its alloys, degradation of titanium-based implants remains clinically relevant, as alterations of the implant surface can influence interactions with both host cells and microorganisms (2, 4–7). Periprosthetic osteolysis is a pathological condition resulting from chronic inflammatory responses induced by implant-derived particulate debris, leading to the recruitment of macrophages, fibroblasts, lymphocytes, and osteoclasts (8–10). Wear debris primarily consists of dissolved metal ions and solid titanium dioxide (TiO_2_) particles, which range from the nanometer to micrometer scale, with particle size and agglomeration state influencing their biological interactions (11–15).

Interactions between periprosthetic cells and TiO_2_ particles are considered a key factor in the development of periprosthetic osteolysis and aseptic implant loosening, as wear particles activate innate immune responses, particularly macrophages, leading to chronic inflammation, reduction of macrophage clearance function, and osteoclast-mediated bone resorption (8, 9, 15). The capacity of titanium wear debris to induce pro-inflammatory signaling has been demonstrated in peri-implant tissues surrounding failed hip prostheses and is supported by growing evidence implicating titanium particles as drivers of inflammatory osteolysis and peri-implant disease (16, 17). Furthermore, analysis of tissues from patients with fatty degenerative osteonecrosis of the jaw (FDOJ) adjacent to titanium dental implants demonstrated dissolved titanium concentrations ranging from 3,200 to 50,600 μg/kg (approximately 0.00032–0.00506 wt%), with the upper end of this range exceeding 0.0025 wt% (25 ppm), a concentration suggested to be biologically relevant for heavy metal particles (18). Likewise, titanium concentrations exceeding 2,000 ppm have been detected in peri-implant soft tissues surrounding orthopedic implants (19–22).

The biological effects of TiO_2_ nanoparticles, including efficient cellular uptake, limited elimination, and persistence within tissues, facilitate prolonged interactions with immune cells (16, 23–25). Among these, macrophages are the principal innate immune cells responsible for recognition and phagocytosis of implant-derived debris and orchestrating inflammatory and reparative responses, that can result in chronic low-grade inflammation complicated by pathological tissue remodeling (8, 10, 26). Owing to their remarkable phenotypic plasticity, macrophages adopt distinct functional programs in response to local microenvironmental cues and are commonly classified into non-activated (M0), classically activated pro-inflammatory (M1), and alternatively activated reparative (M2) subsets, although macrophage activation is now recognized as a functional continuum rather than discrete polarization states (26–28). M1 macrophages contribute to microbial defense and particle clearance through the production of pro-inflammatory cytokines and reactive oxygen species, whereas M2 macrophages promote inflammation resolution, extracellular matrix remodeling, tissue repair, and fibrosis, and are frequently enriched in chronic peri-implant lesions (15, 26, 29–32).

Chitotriosidase (CHIT1) and YKL-40 (CHI3L1) belong to the family of Glyco_18 domain-containing proteins, and are predominantly expressed and secreted by activated and differentiated macrophages. These evolutionarily conserved proteins originated as chitin-binding enzymes involved in host defense against pathogens, although YKL-40 lacks enzymatic activity (33–37). Both CHIT1 and YKL-40 have emerged as biomarkers of chronic inflammation and tissue remodeling in a wide range of inflammatory, fibrotic, and malignant diseases (35, 38–43). Immunohistochemical studies have demonstrated increased YKL-40 expression in fibrotic tissues, suggesting a role in extracellular matrix accumulation and tissue fibrosis (44, 45). Because fibrosis is also a characteristic feature of tissues surrounding titanium implants (26, 46), YKL-40 may be involved in biomaterial-associated tissue remodeling. Supporting a broader role for Glyco_18 proteins in implant-associated responses, increased CHIT1 expression has been reported in periprosthetic tissues from patients with osteolysis and in peri-implant tissues surrounding failed orthopedic implants (17, 46, 47). Together, these findings suggest that YKL-40 and CHIT1 may contribute to macrophage-mediated inflammation and tissue remodeling in response to implanted biomaterials.

Despite increasing evidence that TiO_2_ particles contribute to chronic inflammation following implantation, their effects on CHIT1 and CHI3L1 production by distinct human macrophage subsets remain poorly understood. In the present study, we investigated the effects of TiO_2_ particles on primary human M0, M1, and M2 macrophages. We examined the expression and secretion of CHIT1 and YKL-40 (CHI3L1), assessed CHIT1 enzymatic activity, and evaluated the intracellular localization of CHIT1 following TiO_2_ particle exposure. By characterizing these macrophage-associated Glyco_18 proteins, we aimed to define molecular responses associated with TiO_2_ particle exposure and their potential relevance to biomaterial-associated inflammation.

## 2 Materials and methods

### 2.1 Isolation of human monocytes

Blood monocytes were isolated from buffy coats provided by from German Red Cross Blood Service Baden-Württemberg – Hessen on the commercial basis (48): Buffy coat content was diluted at a 1:1 ratio with phosphate-buffered saline (PBS) without Ca2+- and Mg2+- (Biochrom). Diluted buffy coats containing in total a range of (50 – 100) ml were layered on top several of 15 ml Biocoll (Biochrom, Berlin, Germany) in 50 ml polypropylene tubes followed by centrifugation at temperature at 420 x g without brake for 30 min. Peripheral blood mononuclear cells (PBMC) were collected from the interphase and washed two times with PBS (Biochrom). The PBMCs were further purified by PercollTM density gradient centrifugation at room temperature at 420 x g without break for 30 min. The upper layer, containing monocytes, was collected and washed with PBS for three times. The cells obtained were subjected to CD14+ magnetic cell sorting using monocyte isolation kit (Milteny Biotech, Bergisch Gladbach, Germany), resulting in 95–98% monocyte purity, controlled by flow cytometry. The monocytes were cultured at the concentration of 1 or 2 million cells per ml in customized glucose-free serum-free medium (SFM) supplemented with 5 mM glucose (Life Technology, Darmstadt, Germany), and with cytokines in the presence of 7.5% CO2 for 6 days in presence or in absence of TiO_2_ particles. To induce macrophage differentiation, macrophage colony-stimulating factor (M-CSF, 5 ng/mL) was added. For polarization, IFN-γ (100 ng/mL) was added to generate M1 macrophages, whereas IL-4 (10 ng/mL) was added to generate M2 macrophages (all cytokines were purchased from PeproTech, distributed by TEBU-BIO, Frankfurt am Main, Germany). No cytokines were added for M0 differentiation. In addition, the cells supplemented with 1x10E–8 M of Dexamethasone (Sigma, Munich, Germany) and were maintained in 12 or 24 well-culture dishes.

### 2.2 Preparation of TiO_2_ particles

TiO_2_ (rutile) particles derived from a nanoparticulate starting material (15 wt% dispersion; nominal primary particle size, 15 nm; NanoAmor Europe, France) were used in the experiments. The 15 wt% stock corresponds to a nominal TiO_2_ concentration of 150,000 ppm. The stock suspension was initially diluted 1:4 in DPBS, yielding an intermediate concentration of 37,500 ppm. This intermediate suspension was subsequently diluted in SFM supplemented with 5 mM glucose at 1:1500, 1:750, 1:375, and 1:187.5 to obtain final TiO_2_ concentrations of 25, 50, 100, and 200 ppm, respectively. These concentrations correspond to overall dilution factors of 1:6000, 1:3000, 1:1500, and 1:750 from the original 15 wt% stock. The prepared TiO_2_ suspensions were exposed to UV for 1 h before use.

### 2.3 Physicochemical characterization of TiO_2_ particle agglomeration

The same TiO_2_ material used in the biological experiments was characterized by laser diffraction using a SALD-7101 particle size analyzer (Shimadzu, Kyoto, Japan). Three independent TiO_2_ preparations were prepared in SFM supplemented with 5 mM glucose under closely related medium conditions and incubated for 4–4.8 h before analysis. The preparations were introduced into approximately 200 mL of measurement buffer prepared from a buffer tablet and analyzed under continuous circulation. The original records do not provide the chemical composition of this measurement buffer. Particle-size distributions were recorded before ultrasonication, during ultrasonication at 40 kHz and 40 W, and after termination of ultrasonication at 1, 3, 5, 7, and 9 min. Ultrasonication and continuous circulation were components of the laser diffraction characterization procedure and were not applied during macrophage exposure. Particle-size distributions were evaluated as volume-weighted distributions, and median and mean particle diameters were obtained from the SALD-7101 output. The corresponding particle-size distribution profiles are presented in **Supplementary Figure 1**.

### 2.4 Cellular metabolic activity, YKL-40 secretion, and CHIT1 activity

Cellular metabolic activity was assessed using the Alamar Blue™ Cell Viability Reagent (Thermo Fisher Scientific, Darmstadt, Germany) according to the manufacturer’s instructions in M0, M1, and M2 macrophages cultured in 96-well plates (R&D Systems) in the presence of 0, 25, 50, 100, or 200 ppm TiO_2_ particles. Fluorescence was measured according to the manufacturer’s instructions. YKL-40 (CHI3L1) concentrations in culture supernatants were determined by ELISA (R&D Systems, Wiesbaden, Germany). Extracellular CHIT1 protein concentrations were quantified in macrophage culture supernatants from four independent donors (n = 4) using the Human CHIT1 SimpleStep ELISA Kit (Abcam, Cambridge, UK), according to the manufacturer’s instructions. The assay has a detection range of 15.625–1,000 pg/mL and a sensitivity of 4.63 pg/mL. Supernatants were diluted 1:640 before analysis. CHIT1 enzymatic activity was measured in culture supernatants from five independent donors (n = 5) using a Fluorometric Chitinase Assay Kit (Sigma-Aldrich, St. Louis, MO, USA). Briefly, 10 μL of supernatant was incubated with 90 μL of 4-methylumbelliferyl *N*-acetyl-β-D-glucosaminide substrate, and fluorescence was measured at 360/450 nm (Ex/Em) after termination with sodium carbonate stop solution. Two donors were shared between the CHIT1 ELISA and enzymatic activity assays.

### 2.5 RNA isolation and cDNA synthesis

For RNA isolation, the cells were lysed in TRK lysis buffer and the RNA was isolated using E.Z.N.A Total RNA kit (Omega Bio-tek, Norcross, USA) according to the recommendations of the manufacturer. Total RNA (~100ng) was treated with RNase free DNase I (Thermo Scientific, Germany). cDNA synthesis was performed using RevertAid RT Reverse Transcription Kit (Thermo Scientific, Germany) with oligo-dT primers according to the recommendations of the manufacturer. The obtained cDNA was diluted 1:5 with DEPC water from Thermo Fisher Scientific and 1µL was used for RT-PCR.

### 2.6 Affymetrix microarray analysis

RNA was tested by capillary electrophoresis on an Agilent 2100 bioanalyzer (Agilent) and high quality was confirmed. Hybridization of probes was done using arrays of human HuGene-1_0-st-type (Affymetrix, High Wycombe, UK). Biotinylated antisense cRNA was then prepared according to the Affymetrix standard labelling protocol with the GeneChip^®^ WT Plus Reagent Kit and the GeneChip^®^ Hybridization, Wash and Stain Kit (both from Affymetrix, Santa Clara, USA). Afterwards, the hybridization on the chip was performed on a GeneChip Hybridization oven 640, then dyed in the GeneChip Fluidics Station 450 and thereafter scanned with a GeneChip Scanner 3000. All of the equipment used was from the Affymetrix-Company (Affymetrix, High Wycombe, UK). All the necessary procedures needed for hybridization and scanning of chips were performed in the Next Generation Sequencing (NGS) core facility of Medical Faculty Mannheim at Heidelberg University.

### 2.7 Quantitative PCR analysis

Levels of mRNA for CHI3L1 and CHIT1 were quantified using TaqMan PCR master mix (Applied Biosystems, Darmstadt, Germany). All ready-made primer mixes were used (Hs00609691_m1 for CHI3L1 and Hs00185753_m1 for CHIT1) ordered from Life Technologies. For 18S rRNA FP: CCATTCGAACGTCTGCCCTAT, RP: TCACCCGTGGTCACCATG, probe: JOE-ACTTTCGATGGTAGTCGCCGTGCCT-BHQ1. Amplification was performed using Light cycler 480 system (Roche Lifesciences). The expression levels of analyzed genes were normalized according to the 18s rRNA. All the samples were normalized to maximum response in each donor.

### 2.8 Immunofluorescence and confocal microscopy

The cells used for immunofluorescence were seeded into 24-well plates on top of the round coverslips (1.5 million cells per well) and were cultured in presence or absence of TiO_2_ particles for 6 days. On day 6 the cells were fixed with 2% and 4% formaldehyde (PFA) and either stored or subjected to immunofluorescence staining. The following primary antibodies were used: rabbit polyclonal anti–chitotriosidase (Thermo Fisher Scientific, Germany), mouse monoclonal anti–EEA-1, mouse monoclonal anti-GM130 (BD Bioscience, Heidelberg, Germany), mouse monoclonal anti-TGN46 (Serotec, Feldkirchen, Germany), mouse anti-Lamp1, and mouse anti-Lamp3 (Santa Cruz Biotechnology, Santa Cruz, CA) and rat IgG2a isotype control (eBiosciences, San Diego, CA). Secondary antibodies were Cy3-conjugated goat anti–rabbit IgG and Alexa488-conjugated goat anti–mouse IgG (Dianova, Germany). In addition, all the samples were stained with the following reagents: DAPI (Roche, Mannheim, Germany) and DRAQ5 (Cell Signaling, Danvers, MA USA). Samples were mounted using DakoCytomation Fluorescent Mounting Medium (DakoCytomation, Hamburg, Germany). Confocal microscopy was performed using a Leica laser scanning spectral confocal microscope, model DM IRE2, equipped with an HCX PL Apo 63 ×/1.32 numeric aperture oil objective (Leica Microsystems, Wetzlar, Germany). Excitation was performed with an argon laser emitting at 488 nm, a krypton laser emitting at 568 nm, and a helium/neon laser emitting at 633 nm. Images were acquired using a TCS SP2 scanner and Leica Confocal software, version 2.5 (both from Leica Microsystems). All 2-color images were acquired using a sequential scan mode. For panel assembly, Adobe Photoshop version 6.0 (Adobe Systems, San Jose, CA) and Corel Draw version 10 (Corel, Ottawa, ON, Canada) were used.

### 2.9 Analysis of peri-implant tissues from patients with orthopedic implants

Frozen peri-implant tissue specimens were obtained from eight patients undergoing removal of titanium fracture fixation plates at the Department of Orthopaedic and Trauma Surgery, University Medical Centre Mannheim, Medical Faculty Mannheim, Heidelberg University. Clinical characteristics, implant localization, indications for implantation, and documented clinical findings are summarized in **Supplementary Table S2**. The study was approved by the Ethics Committee of the Medical Faculty Mannheim, Heidelberg University (Approval No. 2016-573N-MA).

For immunofluorescence analysis, tissue sections were air-dried, washed with PBS containing 0.1% Tween-20 (PBST), blocked with Peroxidase Block (Dako) and 3% bovine serum albumin (BSA), and incubated with rabbit anti-human CD68 antibody (Cell Signaling Technology, #76437) and guinea pig anti-YKL-40 antibody (PSL, #010-2). Rabbit IgG (Millipore, #PP64B) and guinea pig preimmune serum (PSL) served as negative controls. Sections were incubated with Alexa Fluor 488-conjugated goat anti-guinea pig IgG and Cy3-conjugated goat anti-rabbit IgG secondary antibodies (Dianova), counterstained with DAPI, mounted in aqueous mounting medium, and analyzed by confocal microscopy.

For immunohistochemical analysis, adjacent frozen sections were air-dried, washed with PBST, treated with Peroxidase Block (Dako), and blocked with 3% BSA in PBS before incubation for 1.5 h at room temperature with rabbit polyclonal anti-human CHIT1 antibody diluted in 1% BSA/PBS. Following washing, sections were incubated for 45 min with an HRP-conjugated goat anti-rabbit IgG secondary antibody, and immunoreactivity was visualized using AEC+ substrate-chromogen solution (Dako). The reaction was terminated by washing with PBST, sections were counterstained with Mayer’s hematoxylin, mounted using DakoCytomation aqueous mounting medium, and examined by bright-field microscopy.

### 2.10 Statistical analysis

Statistical analyses were performed using GraphPad Prism 10.6.1 (GraphPad Software Inc., USA). Data are presented as mean ± SEM unless otherwise stated. For RT-qPCR gene-expression data, paired comparisons between untreated and TiO_2_ particle-treated macrophages were analyzed using the Wilcoxon matched-pairs signed-rank test. For matched experimental data containing multiple conditions, differences were analyzed using the Friedman test followed by Dunn’s multiple-comparisons test, with treated conditions compared with the corresponding untreated control. A *p*-value < 0.05 was considered statistically significant. Significance levels are reported as: *p ≤ 0.05, **p ≤ 0.01 and ***p ≤ 0.001; ns, not significant.

## 3 Results

### 3.1 Physicochemical characterization of TiO_2_ particles

Laser diffraction analysis of three independent TiO_2_ preparations demonstrated substantial preparation-dependent and time-dependent variability in particle-size distributions under the characterization conditions (**Supplementary Figure 1**; **Supplementary Table S1**). Particle-size distributions were evaluated as volume-weighted distributions. Preparation 1 showed submicron distributions during the initial measurements without ultrasonication (D50 0.187–0.192 µm; Dmean 0.209–0.215 µm), followed by micron-scale distributions at later time points (D50 193–201 µm; Dmean 193–200 µm). Preparation 2 showed a predominantly micron-scale distribution without ultrasonication (D50 218 µm; Dmean 217 µm), whereas Preparation 3 showed micron-scale distributions (D50 81–86 µm; Dmean 82–87 µm).

During ultrasonication, submicron distributions were observed in all three preparations, with D50 ranges of 0.180–0.215 µm, 0.151–0.184 µm, and 0.015–0.222 µm for Preparations 1, 2, and 3, respectively; the corresponding Dmean ranges were 0.185–0.228 µm, 0.155–0.189 µm, and 0.016–0.236 µm. Following termination of ultrasonication, micron-scale distributions reappeared in Preparations 1 and 3, whereas Preparation 2 remained in the submicron range throughout the recorded post-ultrasonication period. The very small values measured for Preparation 3 during ultrasonication approached the lower measurement range of the instrument and should therefore be interpreted with caution. Overall, these measurements demonstrate substantial variability in the agglomeration state of the TiO_2_ preparations under the characterization conditions.

### 3.2 TiO_2_ particles do not reduce metabolic activity of differentially activated primary human macrophages

To investigate the effects of TiO_2_ particles on inflammatory responses in the peri-implant microenvironment, we used our previously established primary human monocyte-derived macrophage model system (15, 26, 49). To determine the effects of TiO_2_ particles on cellular metabolic activity, freshly isolated monocytes were differentiated into M0, M1, and M2 macrophages and exposed to increasing TiO_2_ particle concentrations (25, 50, 100, and 200 ppm). Metabolic activity was assessed using the Alamar Blue™ assay after 6 days (**Figure 1**). Alamar Blue fluorescence was normalized separately for each macrophage subtype, with the mean fluorescence of untreated M0, M1, and M2 macrophages defined as 100% for their respective groups. Statistical comparisons were performed against the corresponding untreated control within each macrophage subtype. Overall, metabolic activity showed an increasing trend following TiO_2_ particle exposure, with no reduction in Alamar Blue signal observed across the tested concentrations. A statistically significant increase in metabolic activity was observed in M0 macrophages exposed to 100 ppm TiO_2_, whereas no significant differences were detected at the other concentrations or in M1 and M2 macrophages. These findings indicate that TiO_2_ particle exposure was associated with maintained or increased metabolic activity under the tested conditions. Based on these findings, 100 ppm was selected for subsequent experiments as a concentration associated with a significant metabolic response in M0 macrophages without an observed reduction in metabolic activity in the other macrophage subtypes.

**Figure 1:**
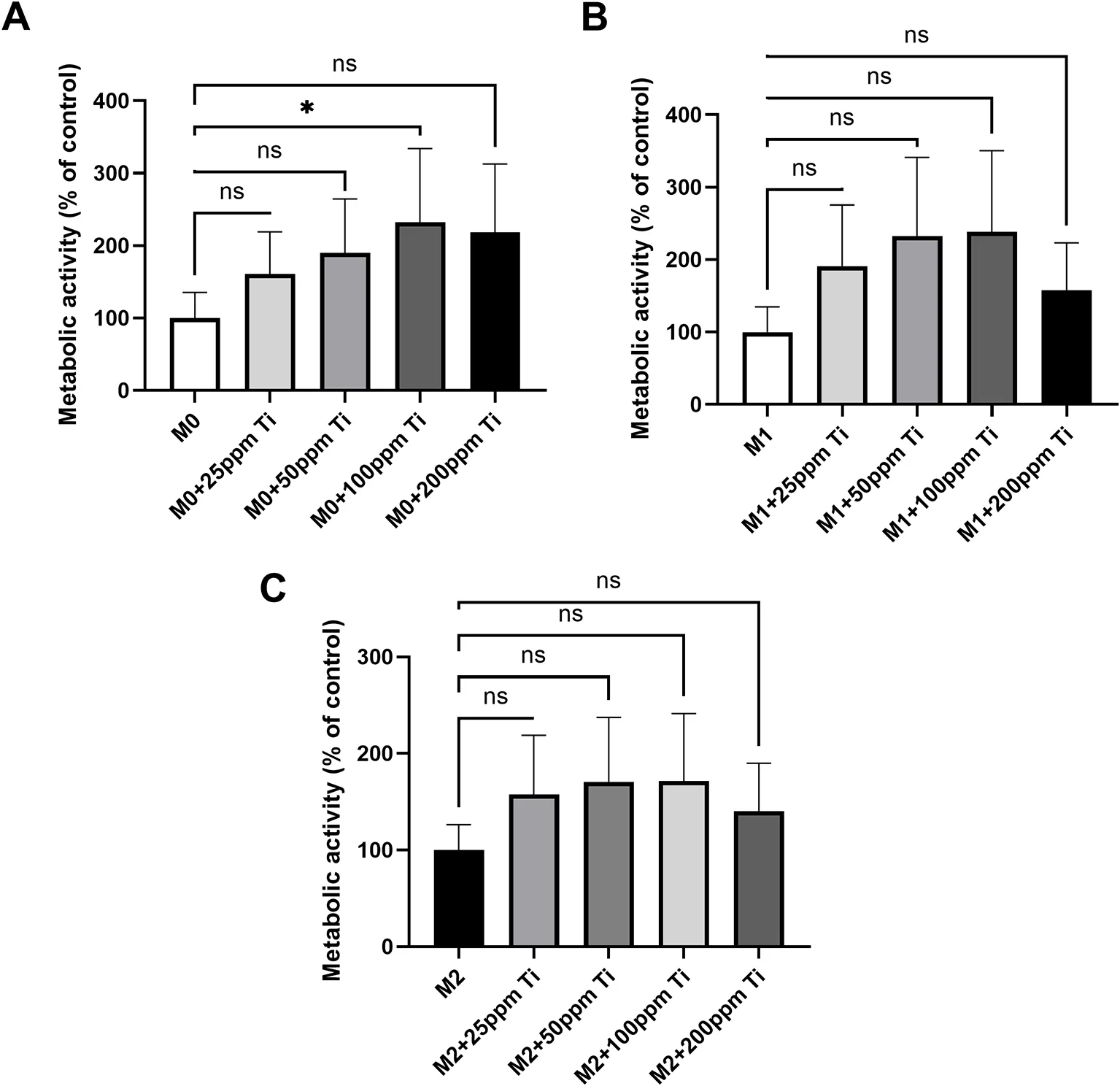
Dose-dependent effect of TiO_2_ particle concentration on metabolic activity of monocyte-derived macrophages. Metabolic activity was assessed using the Alamar Blue assay in unstimulated M0 **(A)**, IFN-γ-stimulated M1 **(B)**, and IL-4-stimulated M2 **(C)** macrophages cultured for 6 days in the absence (0 ppm) or presence of 25, 50, 100, or 200 ppm TiO_2_ particles. Alamar Blue fluorescence was normalized separately for each macrophage subtype, with the mean fluorescence of untreated M0, M1, and M2 macrophages defined as 100% for their respective groups. Data are presented as mean ± SEM from four independent donors (n = 4). Statistical significance was assessed using the Friedman test followed by Dunn’s multiple-comparisons test, comparing each TiO_2_-treated condition with the corresponding untreated control within its macrophage subtype. A statistically significant increase in metabolic activity was observed in M0 macrophages at 100 ppm TiO_2_. *p < 0.05; ns, not significant.

Transcriptome analysis of macrophage responses to TiO_2_ particles.

Microarray analysis demonstrated that exposure to 100 ppm TiO_2_ particles markedly altered macrophage gene expression. A total of 5,098 genes were differentially expressed in M1 macrophages and 4,380 genes in M2 macrophages, with 1,980 genes commonly regulated in both populations (**Figures 2A, B**).

**Figure 2:**
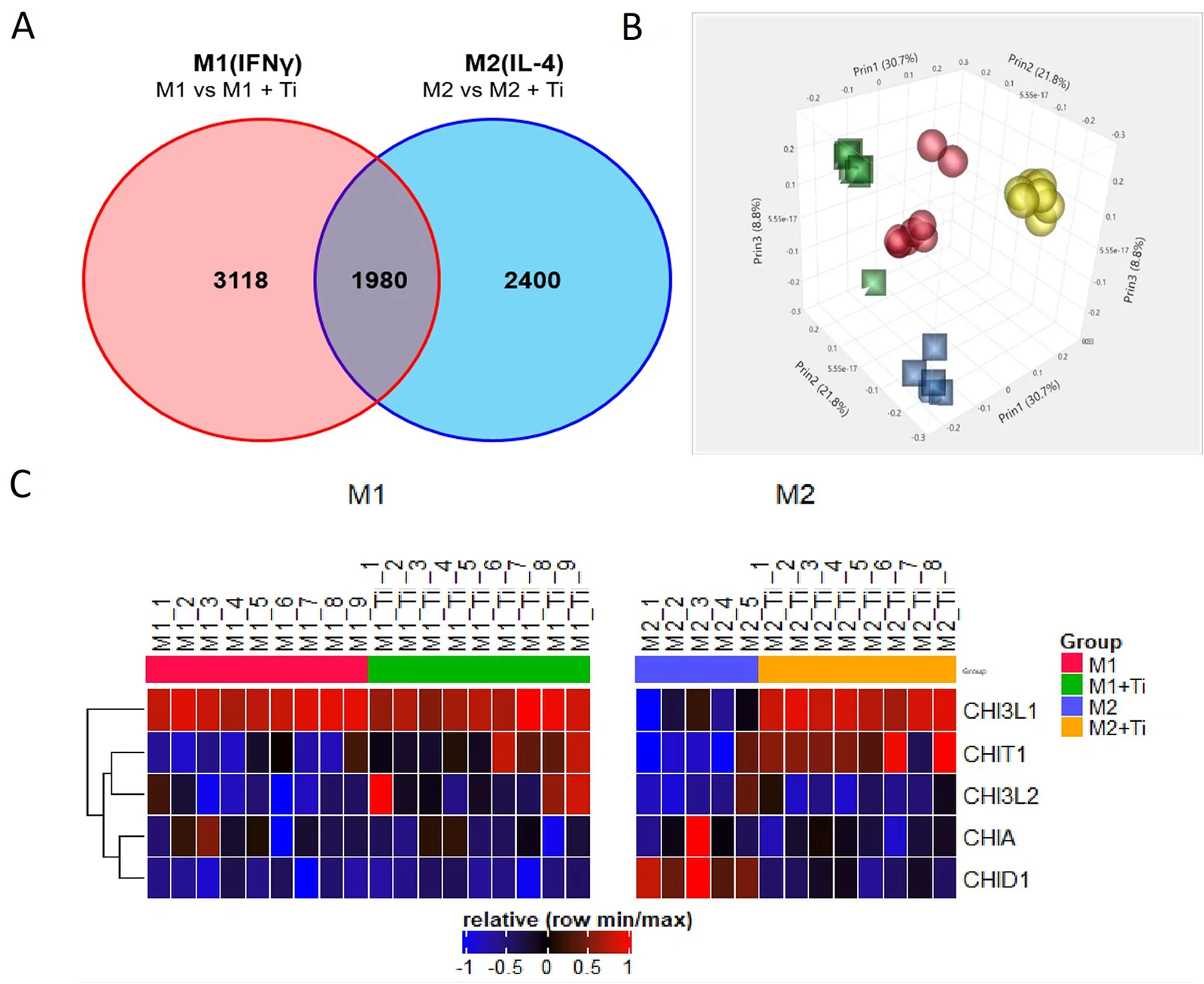
Transcriptome analysis of TiO_2_ particle-induced gene expression in human macrophages. **(A)** Venn diagram showing the numbers of genes differentially expressed in response to 100 ppm TiO_2_ particles in M1 and M2 macrophages. The overlapping region indicates genes commonly regulated in both macrophage subsets. **(B)** Three-dimensional scatter plot illustrating clustering of microarray expression profiles. M1 control and TiO_2_ particle-treated samples are shown in yellow and red, respectively, whereas M2 control and TiO_2_ particle-treated samples are shown in blue and green, respectively. Each data point represents one independent donor. **(C)** Heatmap showing the expression of selected Glyco_18 domain-containing genes in M1 and M2 macrophages cultured in the absence or presence of TiO_2_ particles. The number of independent donors varied between experimental groups according to sample availability.

To identify candidate mediators of TiO_2_ particle-induced immune responses, the expression of five Glyco_18 domain-containing genes (CHIT1, CHI3L1, CHI3L2, CHIA, and CHID1) was evaluated (**Figure 2C**). Among this gene family, CHIT1 and CHI3L1 showed the strongest induction following TiO_2_ particle exposure and were therefore selected for further investigation. CHIT1 expression increased by 2.79-fold in M1 macrophages and 4.76-fold in M2 macrophages. In contrast, CHI3L1 expression was significantly increased only in M2 macrophages (2.35-fold), whereas no significant induction was detected in M1 macrophages.

### 3.3 TiO_2_ particles upregulate gene expression of CHI3L1 and CHIT1 in inflammatory and healing types of macrophages

To validate the microarray findings, CHIT1 and CHI3L1 mRNA expression was quantified by RT-qPCR in freshly isolated monocytes (day 0) and in M0, M1, and M2 macrophages differentiated for 6 days in the absence or presence of 100 ppm TiO_2_ particles (**Figure 3**). No detectable expression of either CHIT1 or CHI3L1 was observed in monocytes. TiO_2_ particle exposure significantly increased CHI3L1 expression in M0 and M2 macrophages (**Figure 3A**), whereas the increase observed in M1 macrophages did not reach statistical significance. The analysis included samples from five donors for M0 and M2 macrophages and eleven donors for M1 macrophages. CHIT1 expression was significantly upregulated by TiO_2_ particles in all macrophage subsets (**Figure 3B**). These data confirm that TiO_2_ particles induce transcriptional activation of CHIT1 in M0, M1, and M2 macrophages and CHI3L1 in M0 and M2 macrophages.

**Figure 3:**
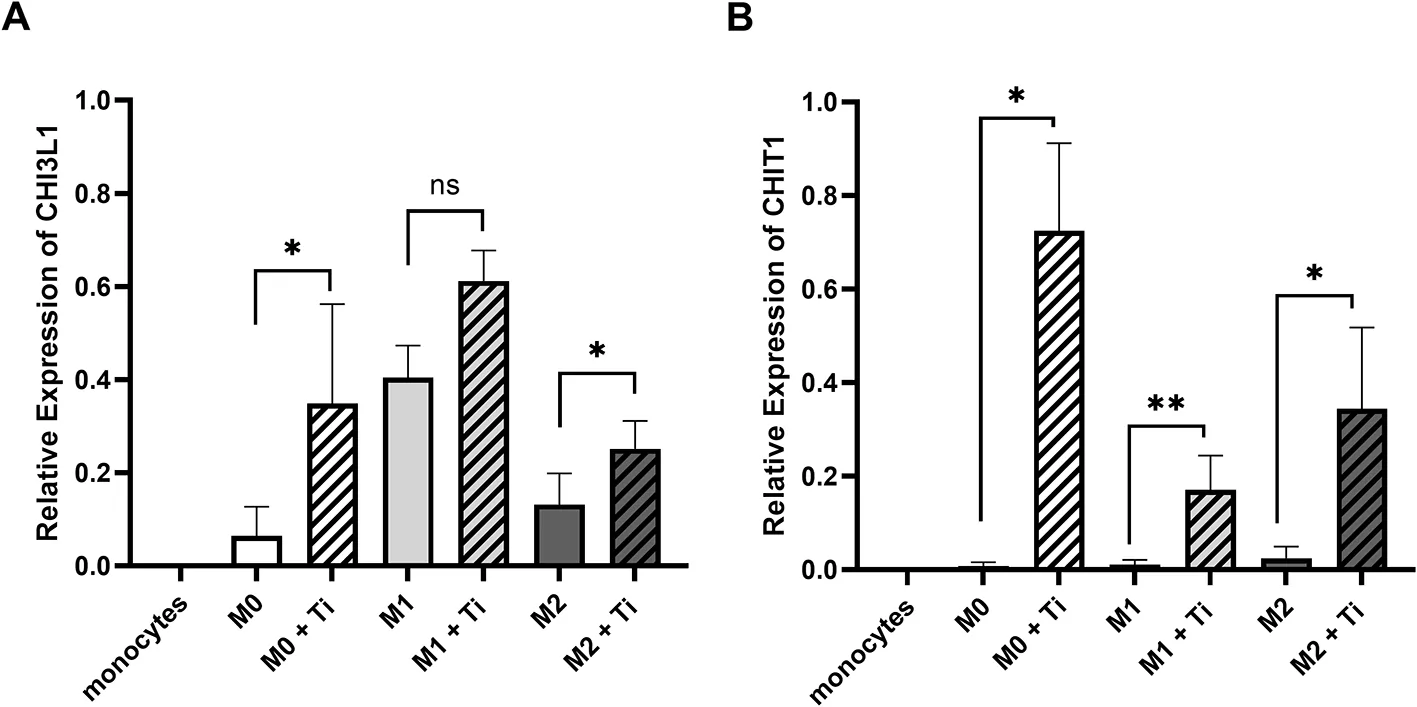
TiO_2_ particles upregulate CHI3L1 and CHIT1 expression in human macrophages. CHI3L1 and CHIT1 mRNA expression was quantified by RT-qPCR in M0, M1, and M2 macrophages cultured for 6 days in the absence or presence of 100 ppm TiO_2_ particles. **(A)** Relative expression of CHI3L1 in M0, M1, and M2 macrophages. **(B)** Relative expression of CHIT1 in M0, M1, and M2 macrophages. Expression values were normalized to the maximum response within each donor. Data are presented as mean ± SEM from 5–11 independent donors, depending on the macrophage subset. Statistical significance was assessed using the Wilcoxon matched-pairs signed-rank test. *p < 0.05; **p < 0.01; ns, not significant. Adapted from Silva-Bermudez et al., 2021 (15).

### 3.4 TiO_2_ particles promote secretion of CHI3L1 in dose dependent manner

To determine whether the transcriptional induction of CHI3L1 resulted in increased protein secretion, YKL-40 concentrations were measured in culture supernatants of M0, M1, and M2 macrophages following exposure to increasing concentrations of TiO_2_ particles (**Figure 4**). YKL-40 secretion increased with increasing TiO_2_ particle concentrations in all macrophage subsets, although the magnitude of the response differed among macrophage phenotypes. M1 macrophages showed the highest absolute YKL-40 concentrations, increasing from an average of approximately 102 ng/mL in untreated cells to 491 ng/mL at 200 ppm TiO_2_ particles, corresponding to an approximately 4.8-fold increase. M0 and M2 macrophages exhibited lower basal YKL-40 concentrations but greater relative responses, increasing approximately 33.8-fold and 63.2-fold, respectively, at 200 ppm compared with untreated controls. Statistically significant increases in YKL-40 secretion compared with untreated controls were observed from 100 ppm TiO_2_ onwards (**Figure 4**). Overall, these findings demonstrate a concentration-dependent increase in YKL-40 secretion following TiO_2_ particle exposure, with distinct response magnitudes among macrophage phenotypes.

**Figure 4:**
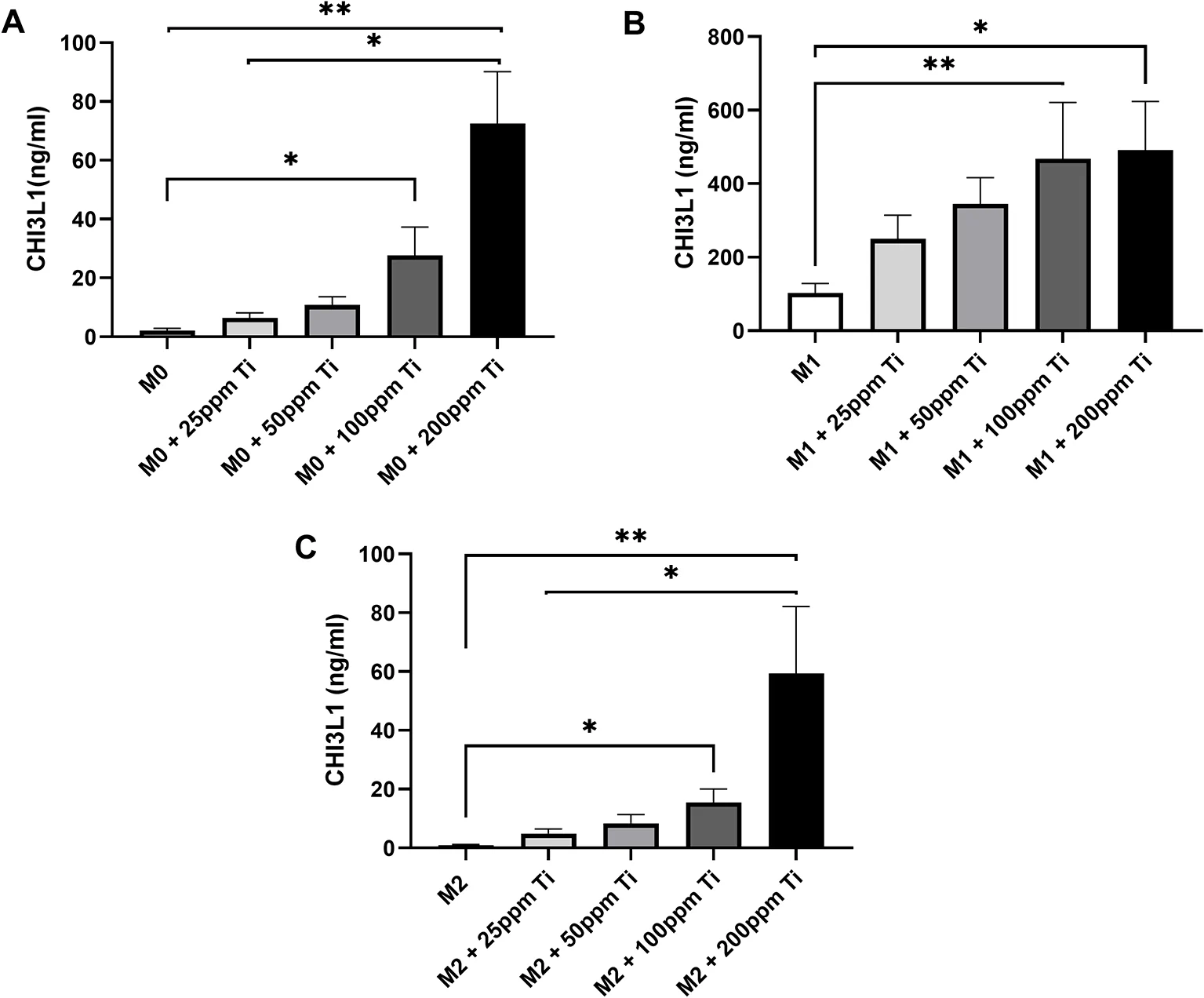
Effect of TiO_2_ particles on YKL-40 (CHI3L1) secretion by human macrophages. YKL-40 concentrations were measured by ELISA in culture supernatants collected from M0, M1, and M2 macrophages after 6 days of culture in the absence or presence of 25, 50, 100, or 200 ppm TiO_2_ particles. **(A)** YKL-40 secretion by M0 macrophages. **(B)** YKL-40 secretion by M1 macrophages. **(C)** YKL-40 secretion by M2 macrophages. Data are presented as mean ± SEM from five independent donors. Statistical significance was assessed using the Friedman test followed by Dunn’s multiple-comparisons test. *p < 0.05; **p < 0.01.

### 3.5 YKL-40 is expressed in peri-implant tissues from patients with titanium orthopedic implants

To determine whether the TiO_2_ particle-induced expression of YKL-40 observed *in vitro* is also present in human peri-implant tissues, specimens obtained from patients undergoing removal of titanium orthopedic fracture fixation implants were analyzed by immunofluorescent staining and confocal microscopy. Tissue samples from eight patients with titanium implants at different anatomical locations were stained for YKL-40 (CHI3L1) and the macrophage marker CD68 (**Figure 5**). YKL-40-positive cells were detected in tissue samples from 5 out of 8 examined patients. Moreover, YKL-40 was expressed in CD68-positive macrophages in patients’ samples, indicating that macrophages represent a major cellular source of YKL-40 within peri-implant tissue microenvironment. Representative images from two male patients with titanium plates of the clavicle and distal tibia are shown in **Figure 5**.

**Figure 5:**
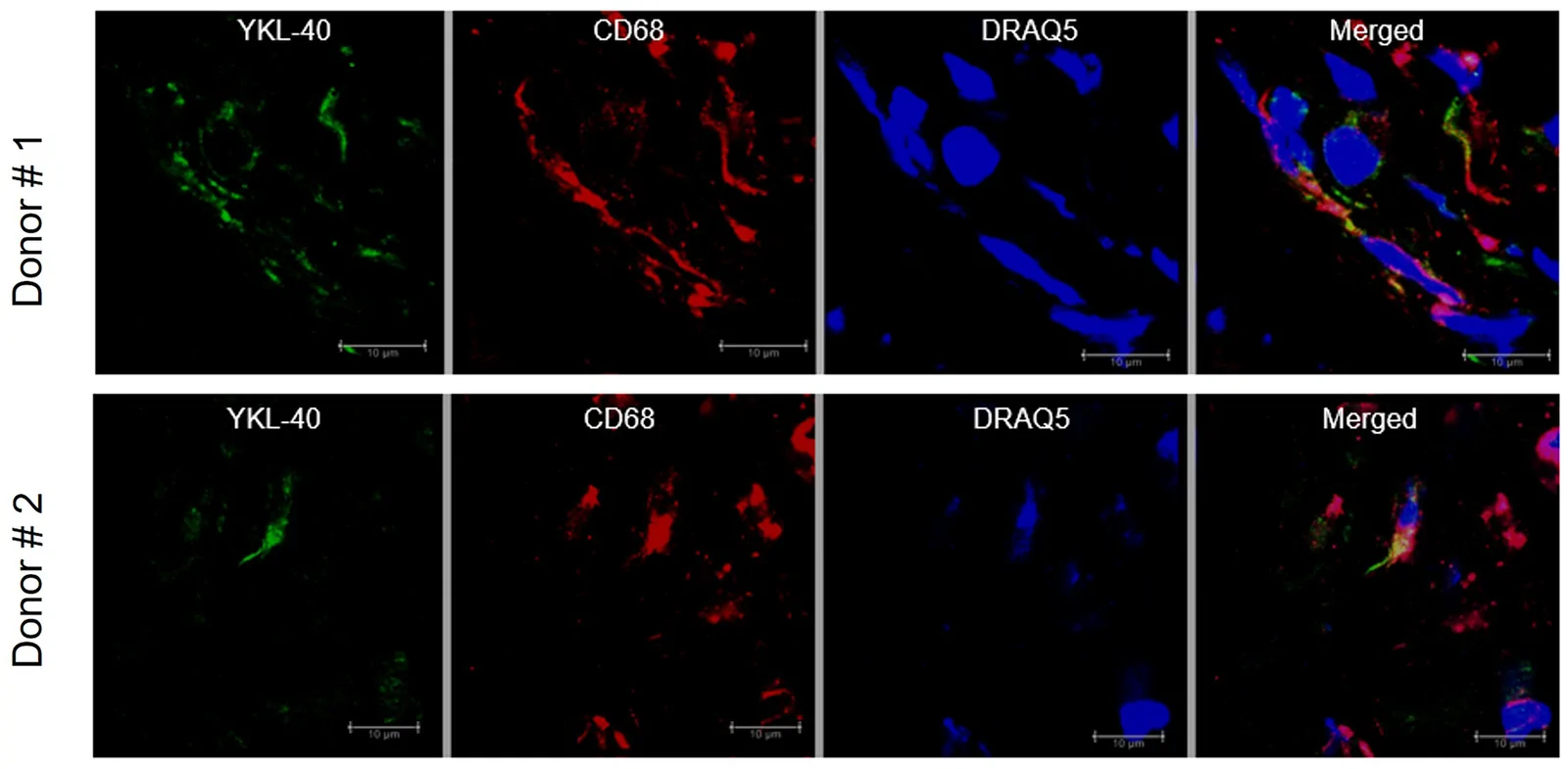
YKL-40 is expressed in CD68+ macrophages in peri-implant tissue. Peri-implant tissue from patients with titanium orthopedic implants was analyzed by immunofluorescence staining for YKL-40 and the macrophage marker CD68. YKL-40 is shown in green, CD68 in red, and nuclei in blue. Yellow signal in the merged images indicates colocalization of YKL-40 and CD68. The upper panel shows tissue located close to the implant, whereas the lower panel shows tissue located farther from the implant. Scale bars, 10 µm.

### 3.6 TiO_2_ particles modulate CHIT1 secretion and activity, and CHIT1 is detected in peri-implant tissues

To determine whether TiO_2_ particle-induced CHIT1 expression was accompanied by changes in extracellular CHIT1 protein abundance and enzymatic activity, CHIT1 protein concentrations and exochitinase activity were measured in culture supernatants from M0, M1, and M2 macrophages exposed to 25 or 100 ppm TiO_2_ particles (**Figure 6**). CHIT1 ELISA showed that 100 ppm TiO_2_ significantly increased extracellular CHIT1 protein concentrations in M0 and M1 macrophages, whereas 25 ppm did not result in a significant increase in any of the macrophage subtypes. In M2 macrophages, neither 25 nor 100 ppm significantly altered extracellular CHIT1 protein concentrations (**Figure 6A**). In contrast, CHIT1 enzymatic activity increased significantly at 25 ppm TiO_2_ in M0 and M2 macrophages, but not in M1 macrophages, whereas no significant increase was detected at 100 ppm despite the increased extracellular CHIT1 protein concentrations observed in M0 and M1 macrophages (**Figure 6B**). These findings demonstrate a discrepancy between extracellular CHIT1 protein abundance and measured enzymatic activity at 100 ppm TiO_2_.

**Figure 6:**
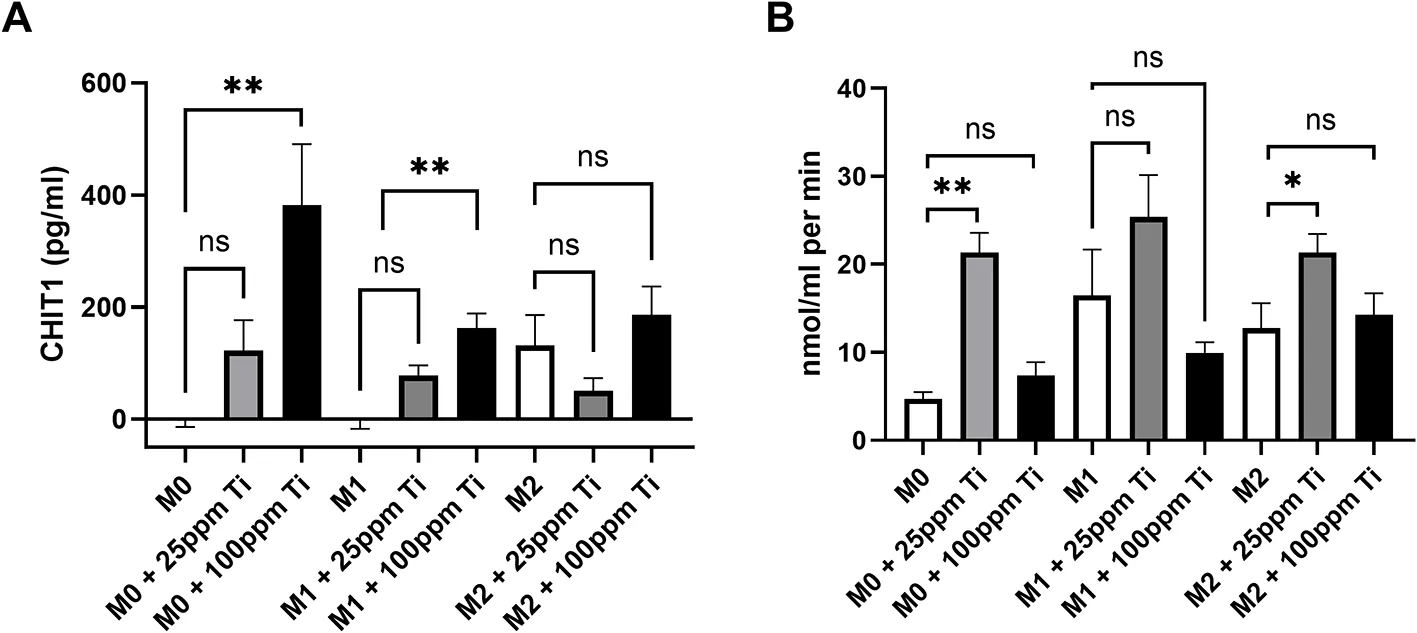
Effect of TiO_2_ particles on extracellular CHIT1 protein abundance and enzymatic activity in human macrophages. **(A)** Extracellular CHIT1 protein concentrations were determined by ELISA in culture supernatants from M0, M1, and M2 macrophages after 6 days of culture in the absence or presence of 25 or 100 ppm TiO_2_ particles (n = 4 independent donors). **(B)** CHIT1 exochitinase activity was measured in culture supernatants under the same experimental conditions (n = 5 independent donors). Data are presented as mean ± SEM. Statistical significance was assessed using the Friedman test followed by Dunn’s multiple-comparisons test. *p < 0.05; **p < 0.01; ns, not significant.

To assess the clinical relevance of these findings, peri-implant tissue samples from five patients with failed titanium orthopedic implants were analyzed for CHIT1 and the macrophage marker CD68 (**Figure 7**). CHIT1-positive cells were detected in three of five tissue specimens and showed colocalization with CD68-positive macrophages. Representative images from two patients with failed titanium clavicular hook plate implants are shown in **Figure 7**.

**Figure 7:**
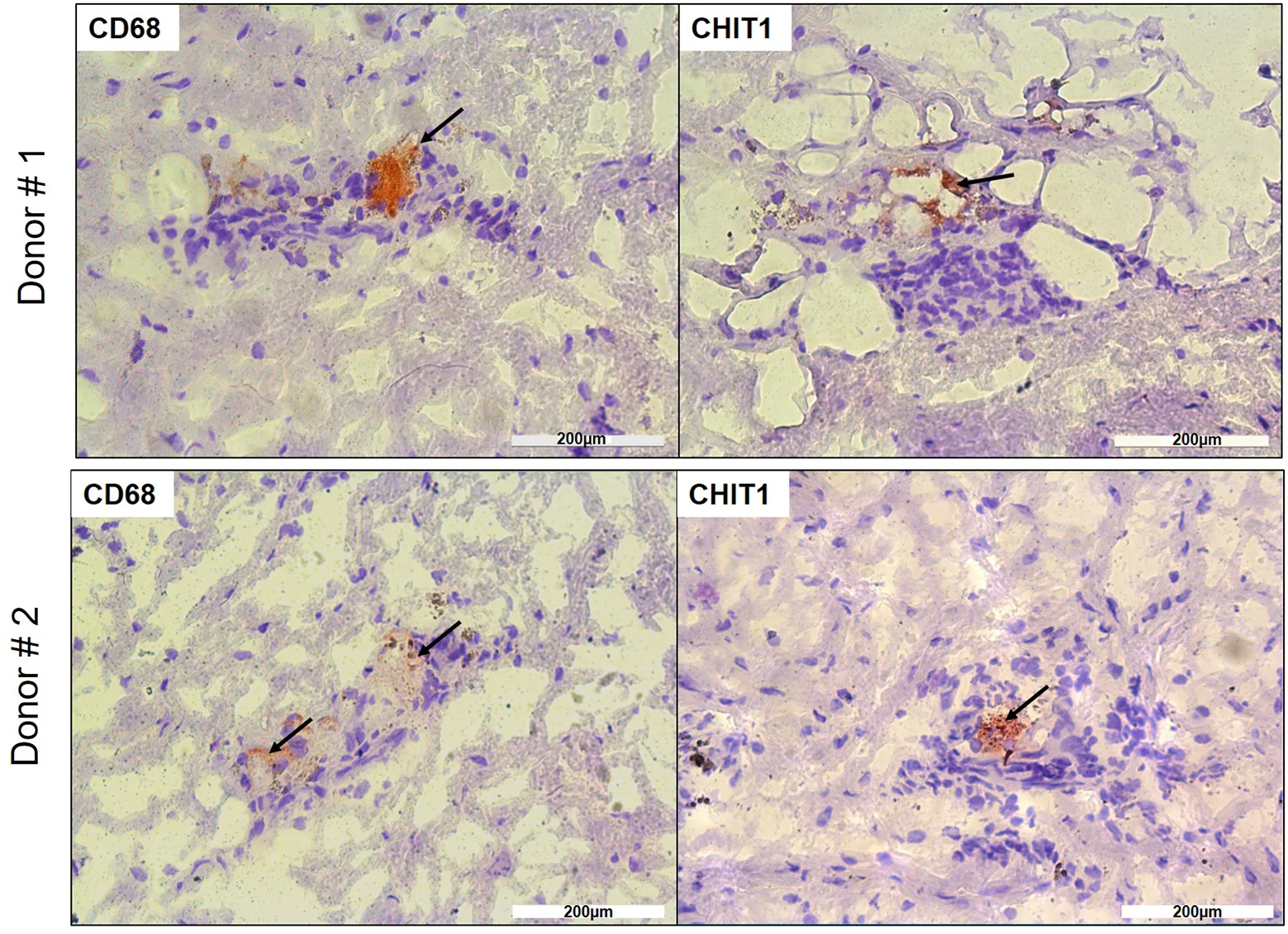
CHIT1 expression in peri-implant tissues from patients with titanium orthopedic implants. Representative immunohistochemical staining of peri-implant tissue sections for CHIT1. Tissue sections were fixed in paraformaldehyde (PFA) and stained using a rabbit polyclonal anti-CHIT1 antibody. Nuclei were counterstained with hematoxylin. Representative images from two patients with failed titanium orthopedic implants are shown. Scale bar, 200 μm.

### 3.7 TiO_2_ particles alter the intracellular localization of CHIT1 in human macrophages

To investigate the intracellular distribution of CHIT1 following TiO_2_ particle exposure, M0, M1, and M2 macrophages were analyzed by confocal immunofluorescence microscopy after 6 days of culture in the absence or presence of 100 ppm TiO_2_ particles (**Figures 8**–**10**).

**Figure 8:**
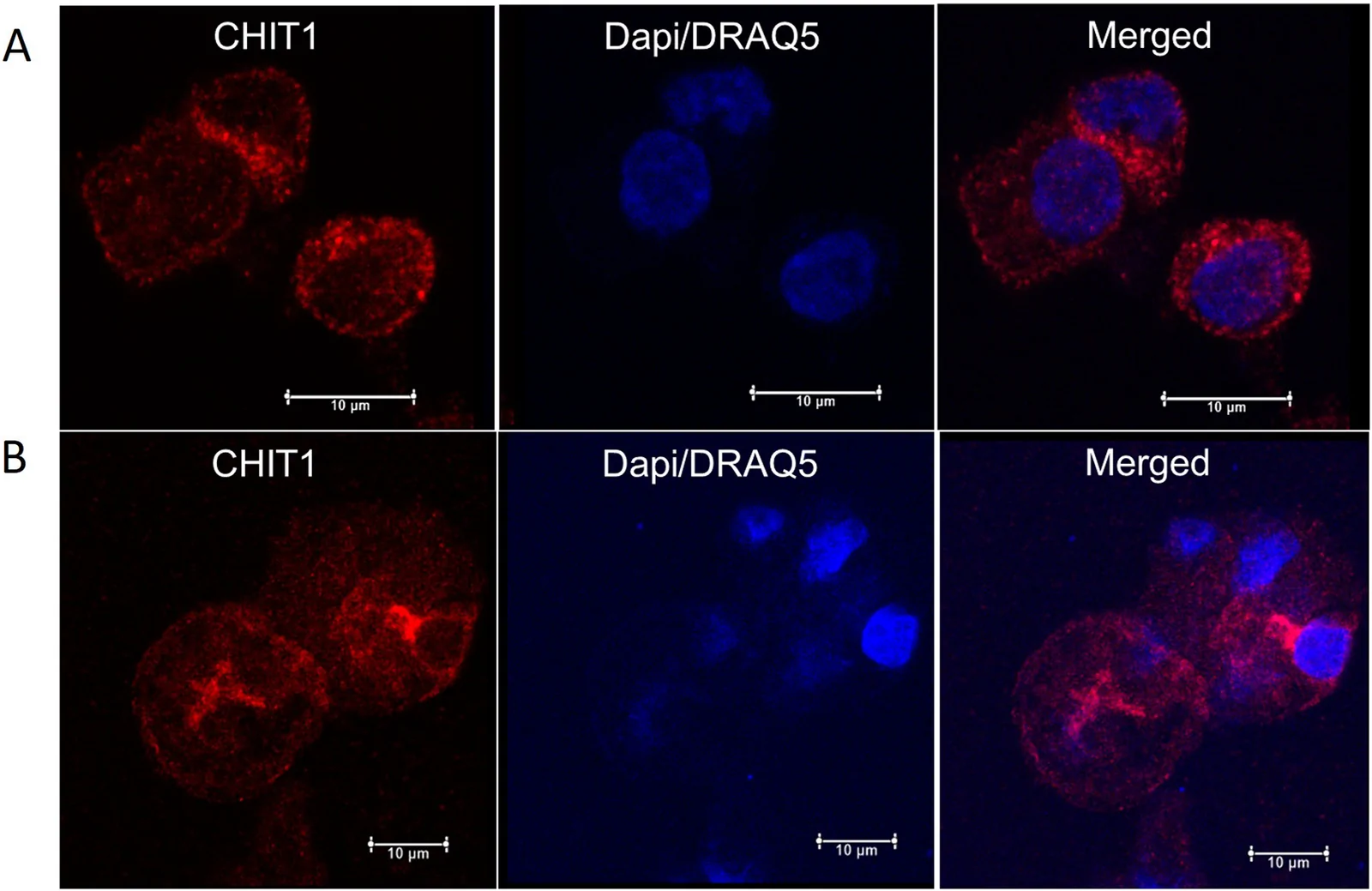
TiO_2_ particles alter the intracellular localization of CHIT1 in M0 macrophages. Representative confocal images of M0 macrophages cultured for 6 days in the absence **(A)** or presence **(B)** of 100 ppm TiO_2_ particles. Cells were fixed with paraformaldehyde (PFA) and stained for CHIT1 (red) using a rabbit polyclonal anti-CHIT1 antibody followed by a Cy3-conjugated goat anti-rabbit IgG secondary antibody. Nuclei were stained with DRAQ5 and DAPI (blue). Scale bars, 10 μm.

**Figure 9:**
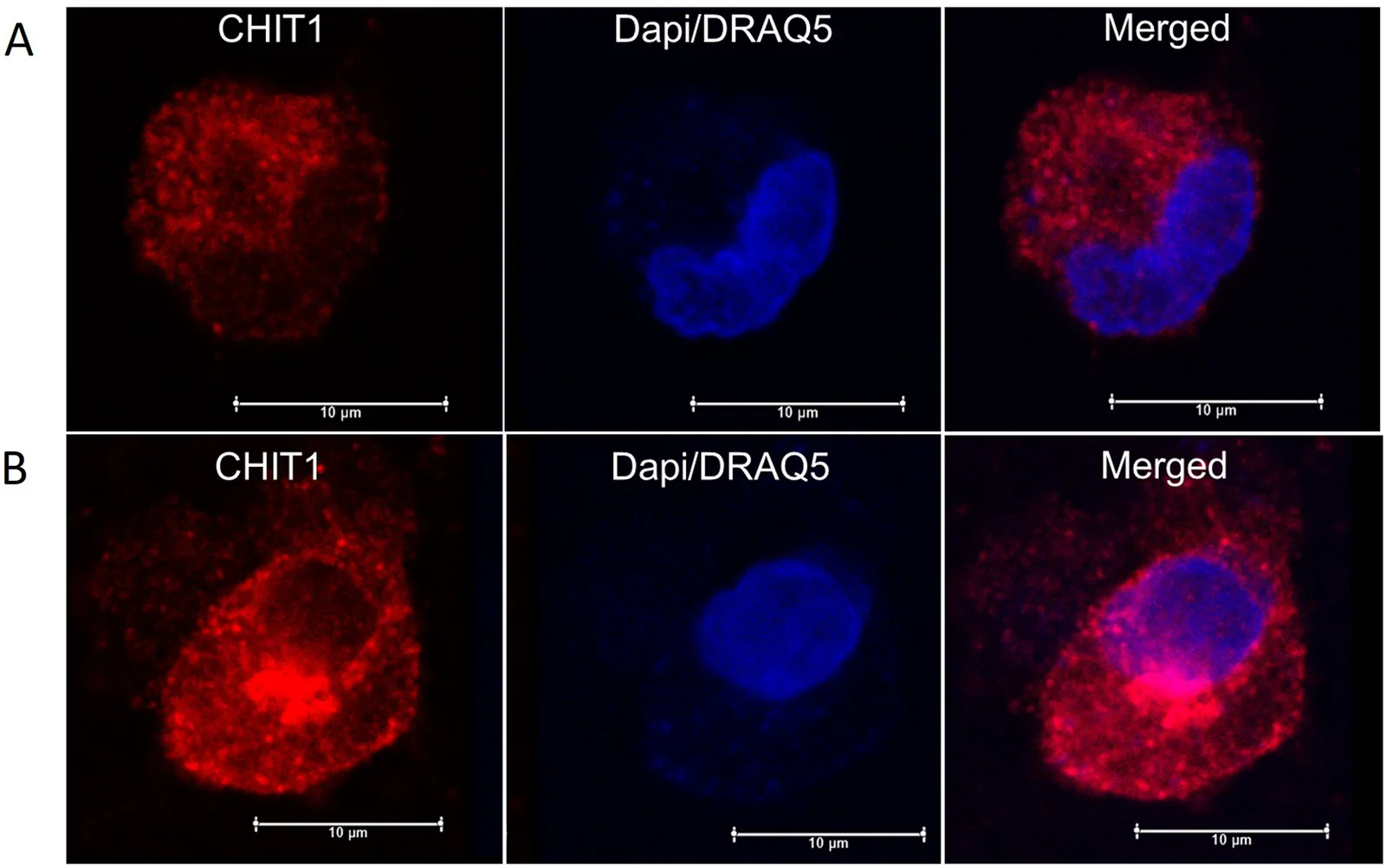
TiO_2_ particles alter the intracellular localization of CHIT1 in M1 macrophages. Representative confocal images of IFN-γ-stimulated (M1) macrophages cultured for 6 days in the absence **(A)** or presence **(B)** of 100 ppm TiO_2_ particles. Cells were fixed with paraformaldehyde (PFA) and stained for CHIT1 (red) using a rabbit polyclonal anti-CHIT1 antibody followed by a Cy3-conjugated goat anti-rabbit IgG secondary antibody. Nuclei were stained with DRAQ5 and DAPI (blue). Scale bars, 10 μm.

**Figure 10:**
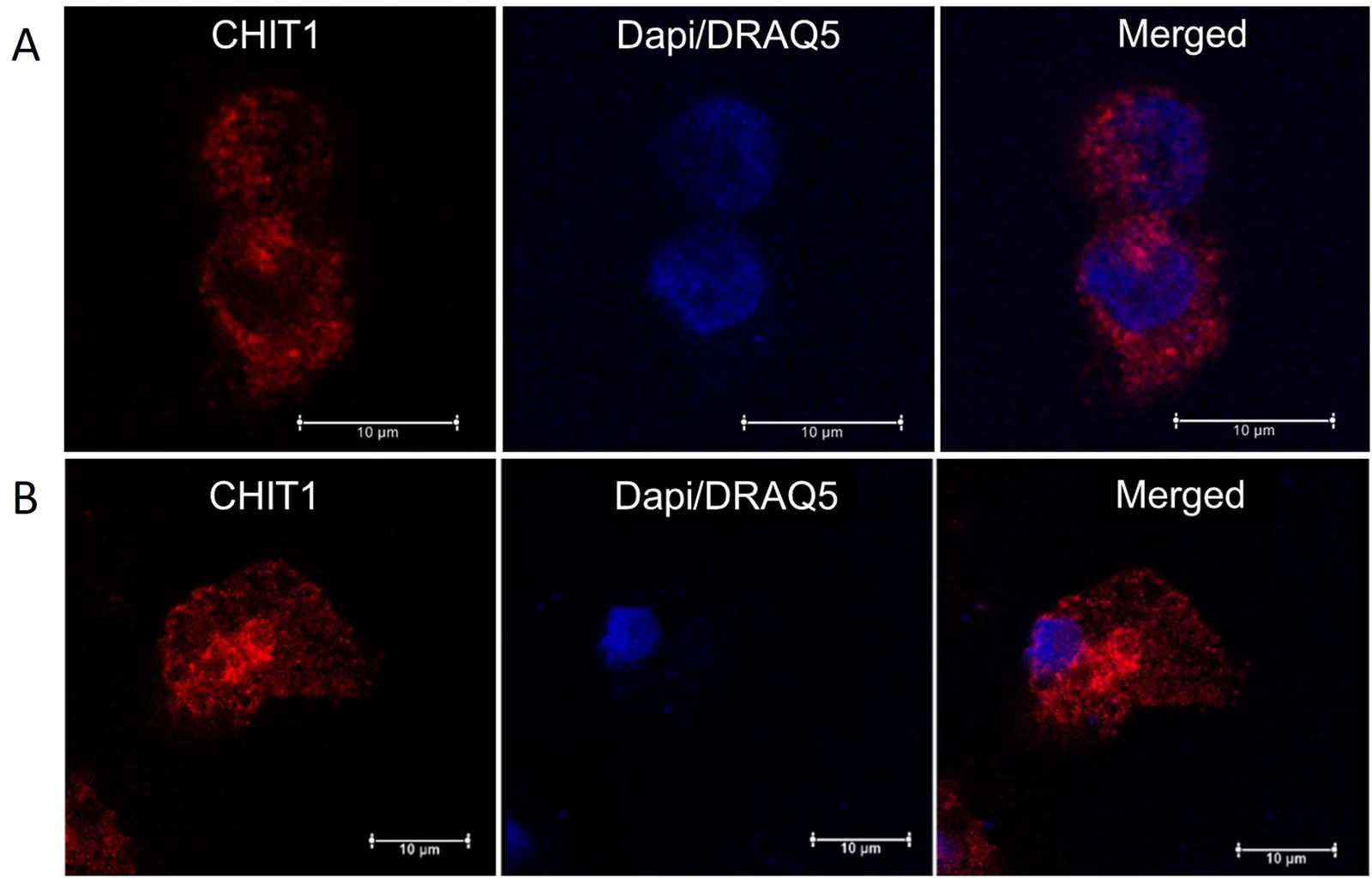
TiO_2_ particles alter the intracellular localization of CHIT1 in M2 macrophages. Representative confocal images of IL-4-stimulated (M2) macrophages cultured for 6 days in the absence **(A)** or presence **(B)** of 100 ppm TiO_2_ particles. Cells were fixed with paraformaldehyde (PFA) and stained for CHIT1 (red) using a rabbit polyclonal anti-CHIT1 antibody followed by a Cy3-conjugated goat anti-rabbit IgG secondary antibody. Nuclei were stained with DRAQ5 and DAPI (blue). Scale bars, 10 μm.

In untreated M0 macrophages, CHIT1 exhibited a diffuse cytoplasmic distribution with weak staining at the plasma membrane (**Figure 8A**). Following TiO_2_ particle exposure, CHIT1 accumulated in distinct intracellular vesicular structures and remained detectable at the plasma membrane, whereas nuclear localization was not observed (**Figure 8B**). In addition, TiO_2_ particle-treated M0 macrophages displayed increased heterogeneity in cell size and morphology.

Untreated M1 macrophages showed weak, diffuse cytoplasmic CHIT1 staining (**Figure 9A**). In contrast, TiO_2_ particle exposure markedly increased CHIT1 expression, with prominent localization at the plasma membrane and within large intracellular vesicles in approximately 40% of the cells analyzed (**Figure 9B**).

Similarly, untreated M2 macrophages exhibited diffuse cytoplasmic CHIT1 staining (**Figure 10A**). Upon TiO_2_ particle exposure, CHIT1 accumulated predominantly within intracellular vesicular structures, although the morphology of these vesicles differed from those observed in M0 and M1 macrophages (**Figure 10B**).

Overall, TiO_2_ particle exposure altered both the abundance and intracellular localization of CHIT1 in all macrophage subsets, suggesting polarization-dependent differences in intracellular organization and potentially trafficking.

### 3.8 TiO_2_ particles disrupt trans-Golgi localization of CHIT1 in human macrophages

To investigate whether the altered intracellular distribution of CHIT1 was associated with changes in the secretory pathway, M0, M1, and M2 macrophages cultured in the absence or presence of 100 ppm TiO_2_ particles were co-stained for CHIT1 and the trans-Golgi network marker TGN46 (**Figure 11**).

**Figure 11:**
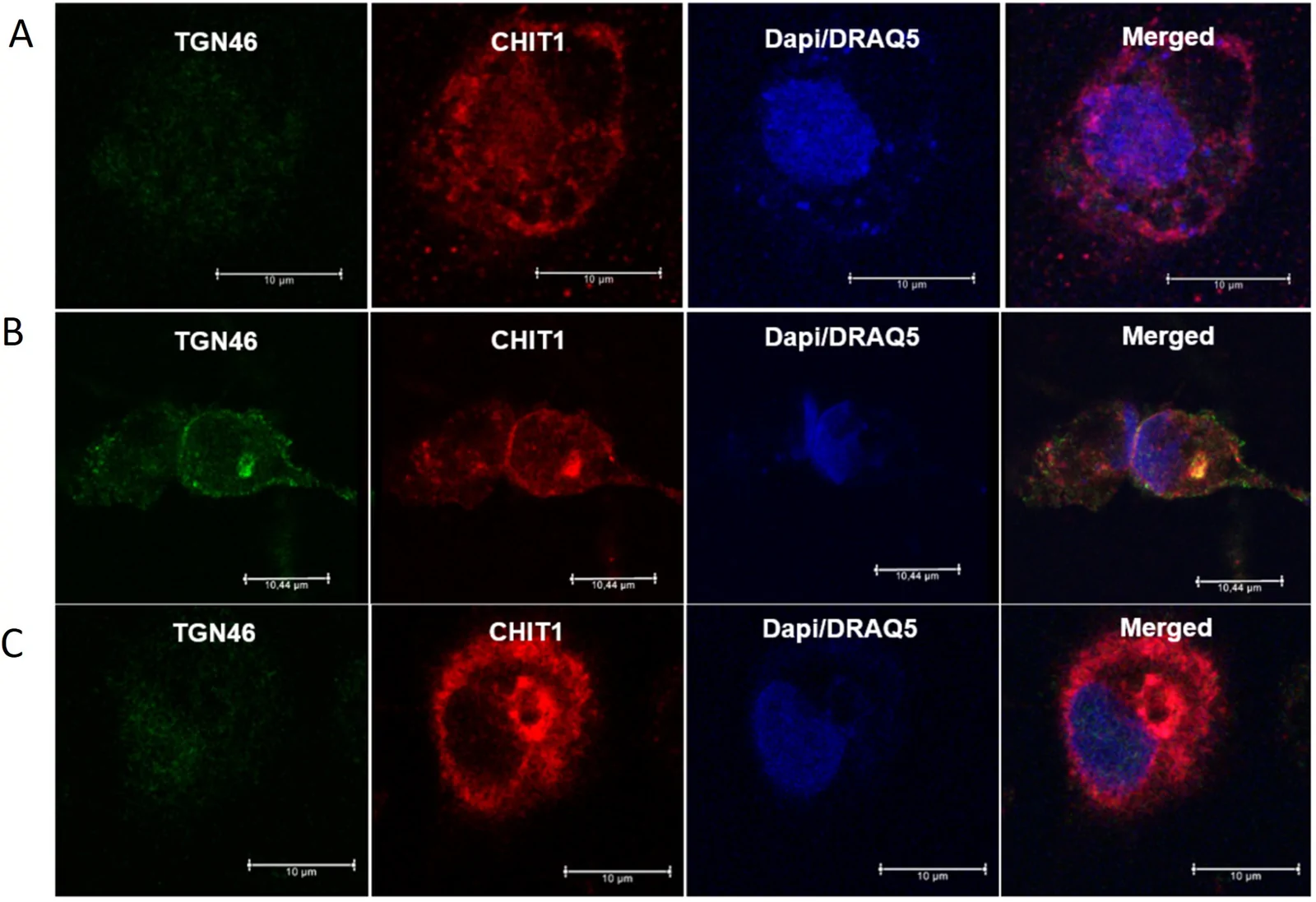
Intracellular localization of CHIT1 and TGN46 in control macrophages. Representative confocal images showing the intracellular localization of CHIT1 and the trans-Golgi network marker TGN46 in macrophages cultured in the absence of TiO_2_ particles (control). Cells were stained for CHIT1 (red) and TGN46 (green), with nuclei counterstained using DRAQ5 and DAPI (blue). Yellow signal in merged images indicates colocalization of CHIT1 and TGN46. **(A)** M0 macrophages, **(B)** IFN-γ-stimulated M1 macrophages, and **(C)** IL-4-stimulated M2 macrophages. Scale bars, 10 μm.

In untreated macrophages, TGN46 staining revealed an intact trans-Golgi network in all macrophage subsets. Following TiO_2_ particle exposure, strong colocalization of CHIT1 with TGN46 was observed in M1 macrophages, indicating accumulation of CHIT1 within the trans-Golgi network and associated vesicular compartments (**Figure 12**). In contrast, TiO_2_ particle-treated M0 and M2 macrophages exhibited marked disruption of the TGN46 staining pattern compared with untreated controls, suggesting disorganization of the trans-Golgi network. In these cells, CHIT1 accumulated predominantly in intracellular vesicular structures without evident colocalization with an intact TGN.

**Figure 12:**
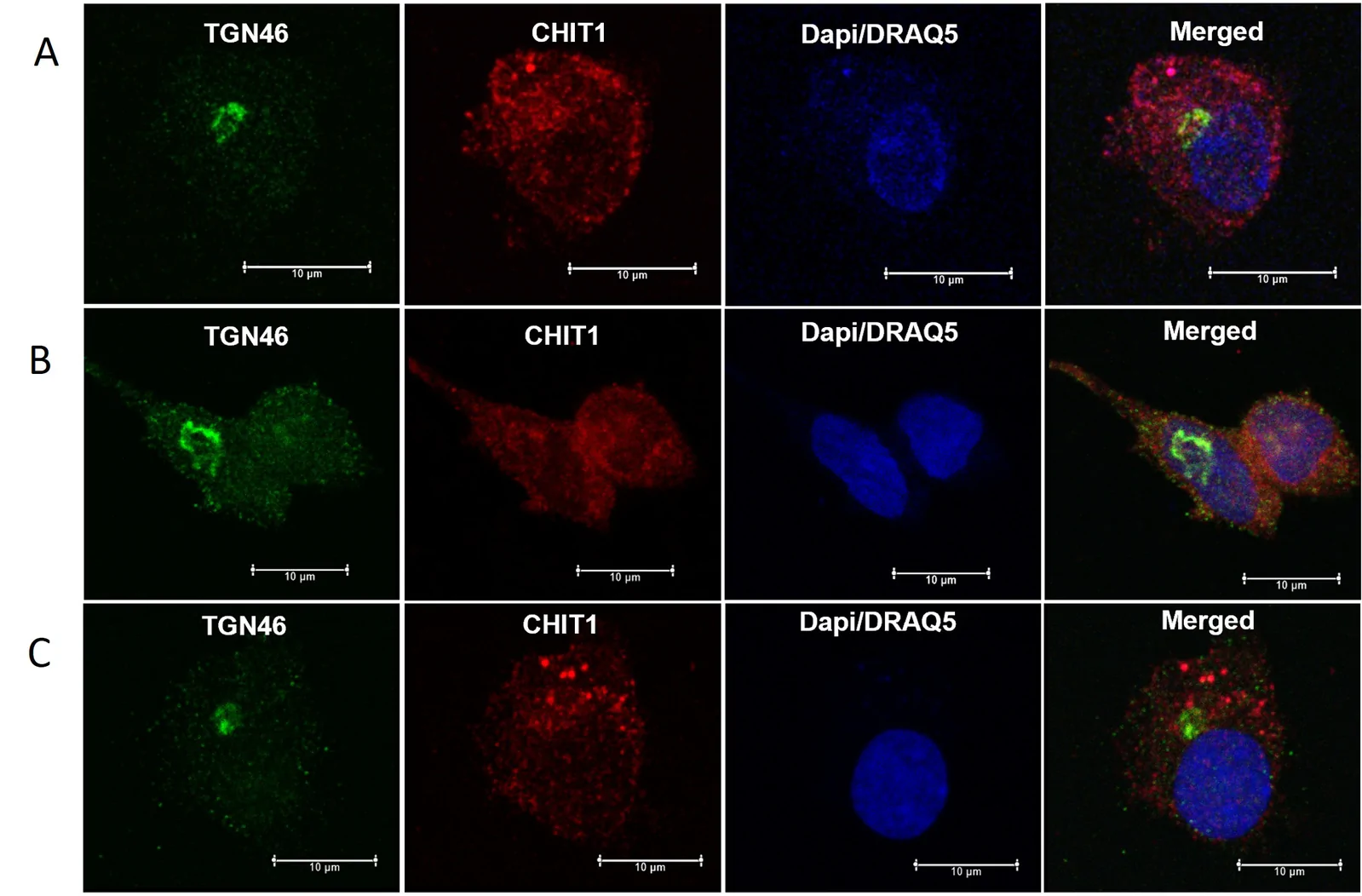
TiO_2_ particles alter the intracellular colocalization of CHIT1 and TGN46 in human macrophages. Representative confocal images showing the intracellular localization of CHIT1 and the trans-Golgi network marker TGN46 in macrophages cultured for 6 days in the presence of 100 ppm TiO_2_ particles. Cells were stained for CHIT1 (red) and TGN46 (green), with nuclei counterstained using DRAQ5 and DAPI (blue). Yellow signal in merged images indicates colocalization of CHIT1 and TGN46. **(A)** M0 macrophages, **(B)** IFN-γ-stimulated M1 macrophages, and **(C)** IL-4-stimulated M2 macrophages. Scale bars, 10 μm.

These findings demonstrate macrophage polarization-dependent differences in CHIT1 localization and trans-Golgi organization following TiO_2_ particle exposure and suggest that these changes may be associated with altered intracellular trafficking.

## 4 Discussion

Our data demonstrate distinct molecular and cellular responses of primary human macrophages to TiO_2_ particle exposure, with metabolic activity assessed at concentrations up to 200 ppm. Alamar Blue measurements, normalized separately to the untreated control of each macrophage subtype, showed an overall trend toward increased metabolic activity following TiO_2_ particle exposure, with no reduction in Alamar Blue signal across the tested concentrations (25, 50, 100, and 200 ppm). A statistically significant increase in metabolic activity was observed in M0 macrophages at 100 ppm TiO_2_, whereas no significant changes were detected at the other concentrations or in M1 and M2 macrophages. Overall, these findings indicate that metabolic activity was maintained or increased under the tested conditions. Similar observations have been reported for macrophages exposed to biomaterials, which exhibit greater resistance to particulate stress than many other cell types owing to their specialized phagocytic function (26, 50–52). Based on these findings, 100 ppm TiO_2_ particles was selected as the concentration for subsequent studies of macrophage responses.

Physicochemical characterization demonstrated substantial agglomeration of the 15-nm rutile TiO_2_ nanoparticulate starting material under the laser diffraction measurement conditions. Because the SALD-7101 provides a volume-weighted (Q_3_) distribution, the measured profiles are strongly influenced by larger agglomerates and do not exclude the presence of a submicron or nanoscale particle fraction. Accordingly, the cellular responses observed in this study should not be interpreted as responses exclusively to freely dispersed 15-nm nanoparticles or exclusively to micron-scale agglomerates. Agglomeration may influence particle sedimentation, cellular contact, and uptake, while larger agglomerates may nevertheless interact with macrophages, including through incomplete or frustrated phagocytosis. Thus, the present findings are interpreted as macrophage responses to TiO_2_ particulate material derived from a nanoparticulate starting material with a pronounced tendency to agglomerate. Laser diffraction demonstrated substantial preparation- and time-dependent variability in particle-size distributions, reflecting variability in the measured agglomeration state under the characterization conditions. Accordingly, no single measured diameter can be considered representative of the TiO_2_ particles throughout the experiment. The very small values obtained for Preparation 3 during ultrasonication approached the lower measurement range of the instrument and should therefore be interpreted cautiously.

Genome-wide transcriptional profiling revealed extensive remodeling of macrophage gene expression following TiO_2_ particle exposure. Among the most strongly induced genes were CHIT1 and CHI3L1, two members of the Glyco_18 protein family that are predominantly expressed by activated macrophages. CHIT1 is the major enzymatically active human chitinase, whereas CHI3L1 (YKL-40) lacks catalytic activity but functions as an important regulator of inflammation, extracellular matrix remodeling, and tissue repair (33, 34, 53). The simultaneous induction of both genes suggests that CHIT1 and CHI3L1 are components of a broader macrophage response to TiO_2_ particle exposure.

The differential regulation of CHIT1 and CHI3L1 among macrophage subsets further highlights the complexity of macrophage responses to implant-derived particles. CHIT1 was induced in all polarization states, with the strongest response observed in M0 macrophages, whereas CHI3L1 was predominantly increased in M2 macrophages. Previous studies have demonstrated that expression of chitinase family members is closely linked to macrophage activation and functional specialization (27, 33–37, 54–56). In particular, CHI3L1 has been implicated in tissue remodeling and fibrosis, while CHIT1 has been associated with chronic inflammatory disorders and macrophage-mediated host defense (34, 35, 38, 57–59). Our findings therefore suggest that TiO_2_ particle exposure is associated with a macrophage innate immune response characterized by increased CHIT1 and YKL-40 expression, which may contribute to persistent peri-implant inflammation.

The pronounced induction of CHIT1 also raises the possibility that TiO_2_ particles engage pattern-recognition pathways normally associated with responses to foreign particulate material. Although CHIT1 has classically been associated with antimicrobial defense, increasing evidence indicates that it also participates in sterile inflammatory responses and macrophage activation (37, 57, 60, 61). Consistent with this concept, Chen et al. demonstrated that TiO_2_ nanoparticles activate Toll-like receptor signaling and promote inflammatory responses in macrophages (62). The marked upregulation of CHIT1 observed in our study supports the hypothesis that TiO_2_ particles are recognized as danger-associated stimuli that initiate innate immune activation.

The marked increase in CHI3L1 transcription was accompanied by a dose-dependent increase in YKL-40 secretion, demonstrating that TiO_2_ particle-induced macrophage activation is translated into functional protein production. While M1 macrophages secreted the highest absolute amounts of YKL-40, the strongest relative induction was observed in M0 and M2 macrophages, indicating that TiO_2_ particles enhance YKL-40 production across different macrophage activation states.

Consistent with our *in vitro* findings, YKL-40+CD68+ macrophages were detected in peri-implant tissues obtained from patients undergoing removal of titanium orthopedic implants, supporting the clinical relevance of our observations. YKL-40 is increasingly recognized as a mediator of chronic inflammation and tissue remodeling produced by activated macrophages (33, 38, 53, 63). Persistent macrophage activation is a hallmark of the foreign body response to orthopedic biomaterials and has been implicated in implant-associated inflammation (5, 50). Our findings therefore identify YKL-40 as a potential marker of macrophage activation in response to titanium particles and support its further investigation in biomaterial-associated inflammation.

Although TiO_2_ particles markedly increased CHIT1 gene expression, this was not consistently accompanied by increased enzymatic activity. At 25 ppm, TiO_2_ particles significantly increased CHIT1 enzymatic activity in M0 and M2 macrophages, whereas no significant increase was observed in M1 macrophages. At 100 ppm, no significant increase in enzymatic activity was detected in any macrophage subtype despite robust transcriptional upregulation. However, ELISA demonstrated significantly increased extracellular CHIT1 protein concentrations at 100 ppm in M0 and M1 macrophages. Thus, the lack of increased enzymatic activity at 100 ppm cannot be explained simply by reduced CHIT1 secretion and instead indicates that extracellular CHIT1 protein abundance and enzymatic activity are not necessarily proportional. This distinction is supported by Yap et al., who assessed CHIT1 enzymatic activity and secreted CHIT1 protein as separate readouts in macrophage culture supernatants (64). CHIT1 also undergoes post-translational processing, with the 50-kDa form being secreted and intracellular processing generating a 39-kDa form that remains enzymatically active (38, 60, 64–66). The precise mechanism underlying the discrepancy observed in our study remains unclear. Differences in CHIT1 processing or enzyme state may contribute. However, particle-associated interference with the fluorometric assay, including protein adsorption or fluorescence quenching, cannot be excluded because cell-free interference controls were not performed.

Importantly, CHIT1+CD68+ macrophages were detected in peri-implant tissues obtained from patients undergoing removal of titanium orthopedic implants, supporting the clinical relevance of our *in vitro* observations. Previous studies have reported increased CHIT1 expression in periprosthetic tissues associated with implant loosening and macrophage activation (8, 17, 47, 67). Together, these findings identify CHIT1 as a macrophage-associated marker of the innate immune response to TiO_2_ particles and support its potential involvement in biomaterial-associated inflammation. However, the TiO_2_ particles used *in vitro* cannot be assumed to fully reproduce the heterogeneous physicochemical characteristics of titanium-containing debris generated *in vivo*, despite the substantial agglomeration observed under our experimental conditions. Other factors associated with implant failure, including infection or mechanical instability, may also contribute to CHIT1 and YKL-40 expression in peri-implant tissues.

In addition to increasing CHIT1 expression, TiO_2_ particles altered its intracellular localization. Whereas CHIT1 displayed a diffuse cytoplasmic distribution in untreated macrophages, TiO_2_ particle exposure promoted its accumulation within intracellular vesicles and, in M1 macrophages, at the plasma membrane. These findings suggest that TiO_2_ particles influence intracellular trafficking pathways in addition to regulating CHIT1 expression.

CHIT1 is synthesized within the secretory pathway and undergoes vesicular trafficking before being either secreted or targeted to lysosomes (60, 68). The distinct localization patterns observed among M0, M1, and M2 macrophages suggests that macrophage polarization may influence CHIT1 intracellular trafficking in response to particulate stimuli. Importantly, altered localization of CHIT1 following TiO_2_ particle exposure does not necessarily imply enhanced secretion. Instead, the discrepancy between increased extracellular CHIT1 protein levels and the limited increase in enzymatic activity at 100 ppm suggests that CHIT1 protein abundance and enzymatic function may be regulated differently following TiO_2_ particle exposure. However, the underlying processes remain to be determined.

Confocal microscopy demonstrated that TiO_2_ particle exposure altered the intracellular trafficking of CHIT1 in a macrophage subtype-dependent manner. Whereas M1 macrophages retained CHIT1 within TGN46-positive compartments, M0 and M2 macrophages exhibited disruption of the trans-Golgi network accompanied by redistribution of CHIT1 into intracellular vesicles. These observations are consistent with previous studies demonstrating that CHIT1 is synthesized within the secretory pathway, traffics through the Golgi apparatus and trans-Golgi network, and undergoes vesicular sorting before secretion or lysosomal targeting (37, 60, 65, 66, 68).

Disruption of Golgi organization has emerged as an important feature of macrophage activation and cellular stress. Recent studies have shown that trans-Golgi disassembly can accompany activation of innate immune pathways, including NLRP3 inflammasome signaling (69–71). Although inflammasome activation was not investigated in the present study, our findings suggest that TiO_2_ particle exposure is associated with alterations in intracellular organization and trafficking-related processes in macrophages. However, the present data do not establish whether these changes contribute to the discordance between increased CHIT1 transcription and the limited increase in extracellular enzymatic activity at higher TiO_2_ particle concentrations. Beyond CHIT1 processing, disruption of trans-Golgi and vesicular trafficking may have broader consequences for macrophage function, as these pathways are involved in the processing and secretion of inflammatory mediators as well as membrane trafficking associated with antigen processing and presentation (69–71). Thus, TiO_2_ particle-induced alterations in TGN organization could potentially affect cytokine secretion and antigen-presenting functions, thereby contributing to persistent macrophage activation in the peri-implant microenvironment. However, these functional consequences were not directly assessed in the present study and therefore require further investigation.

Together, our data identify CHIT1 and YKL-40 as macrophage-associated proteins induced by TiO_2_ particles and demonstrate that TiO_2_ particle exposure influences both their expression and intracellular processing. These findings provide new insight into the macrophage innate immune response associated with titanium particle exposure and support further investigation of chitinase family proteins as potential biomarkers and modulators of biomaterial-associated inflammation. Nevertheless, several limitations should be acknowledged. First, although TiO_2_ particles induced CHIT1 and CHI3L1 expression and altered their activity, secretion, and intracellular localization, functional perturbation was not performed; therefore, their mechanistic role in TiO_2_ particle-induced immune activation remains to be established. Second, although TiO_2_ particle agglomeration was characterized by laser diffraction, the characterization was performed under analytical conditions that differed from the six-day macrophage exposure, including the measurement volume and medium, continuous circulation, duration of analysis, and application of ultrasonication during parts of the characterization procedure. Therefore, the particle-size distributions measured by laser diffraction cannot be assumed to represent the particle-size distribution present throughout the macrophage cultures. In addition, hydrodynamic size and surface charge were not determined. Although the experiments were performed in serum-free medium, adsorption of medium components and cell-secreted proteins to the TiO_2_ particle surface cannot be excluded, and protein-corona formation was not directly characterized. Furthermore, because TiO_2_ surface properties before and after UV sterilization were not characterized, potential UV-induced changes in particle surface reactivity, photocatalytic ROS generation, and modification of medium components cannot be excluded. A particle-free control medium subjected to identical DPBS dilution and UV treatment was not included, representing an additional limitation of the study. Finally, the clinical cohort was small and anatomically heterogeneous; thus, the tissue findings support clinical relevance but do not establish a causal role for CHIT1 or YKL-40 in implant failure. The variable detection of CHIT1 and YKL-40 among specimens may reflect heterogeneity in clinical presentation, implant location, and implantation history. Larger, clinically stratified cohorts will therefore be required to validate CHIT1 and YKL-40 as biomarkers of implant-associated inflammation.

## 5 Conclusions

In conclusion, TiO_2_ particle exposure induces distinct molecular responses in primary human macrophages while maintaining or increasing metabolic activity under the tested conditions, with a significant increase observed in M0 macrophages at 100 ppm. TiO_2_ particles promoted the expression and secretion of the chitinase family members CHIT1 and YKL-40 (CHI3L1), identifying these proteins as macrophage-associated responses to titanium particle exposure. While 25 ppm TiO_2_ particles significantly enhanced CHIT1 enzymatic activity in M0 and M2 macrophages, higher particle concentrations increased extracellular CHIT1 protein abundance in M0 and M1 macrophages without a corresponding significant increase in enzymatic activity, indicating a dissociation between CHIT1 protein abundance and catalytic activity. Furthermore, TiO_2_ particles altered the intracellular localization of CHIT1 and disrupted trans-Golgi organization, consistent with alterations in intracellular organization and trafficking-related processes. Together, these findings provide new insight into macrophage responses to TiO_2_ particles and identify.

## Data Availability

The datasets presented in this study can be found in online repositories. The names of the repository/repositories and accession number(s) can be found below: https://www.ncbi.nlm.nih.gov/, GSE179543.
