# Supplementary material for: TiO2 particles induce CHIT1 and YKL-40 in human macrophages: implications for implant-associated inflammation
Source: Front Immunol. 2026 Oct 28;17:1964504. doi: 10.3389/fimmu.2026.1964504 (PMC13751356; doi:10.3389/fimmu.2026.1964504)
Supplement: Supplementary file 1 [file DataSheet1.pdf]

A

File Name	Before ultrasonication – 9 min	Meas Date	18/05/12 18:34:27
Sample ID		Sample No.	
Comment			

Median D	192.965	Mean V	193.404
Modal D	177.828	Std Dev	0.101

10%D(μm)	50%D(μm)	90%D(μm)	0%D(μm)	0%D(μm)	0%D(μm)	0%D(μm)	0%D(μm)	0%D(μm)
138.788	192.965	269.199	0.000	0.000	0.000	0.000	0.000	0.000

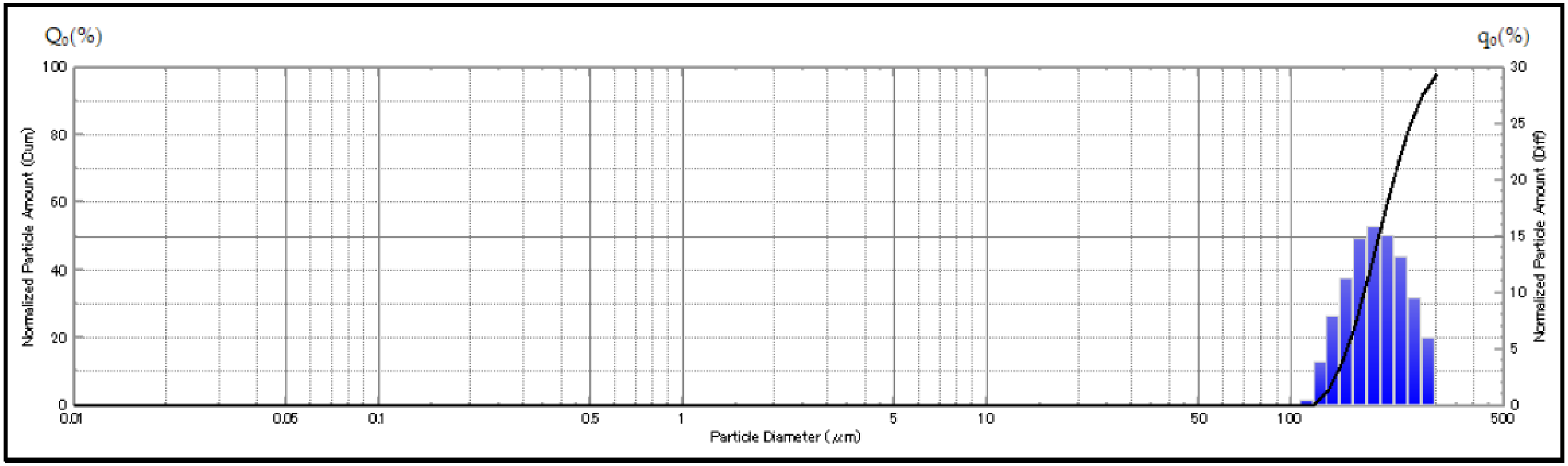

B

File Name	During ultrasonication – 9 min			Meas Date	18/05/12 18:44:26
Sample ID		Sample No.			
Comment					

Median D	0.180	Mean V	0.185
Modal D	0.178	Std Dev	0.114

10%D(μm)	50%D(μm)	90%D(μm)	0%D(μm)	0%D(μm)	0%D(μm)	0%D(μm)	0%D(μm)	0%D(μm)
0.133	0.180	0.265	0.000	0.000	0.000	0.000	0.000	0.000

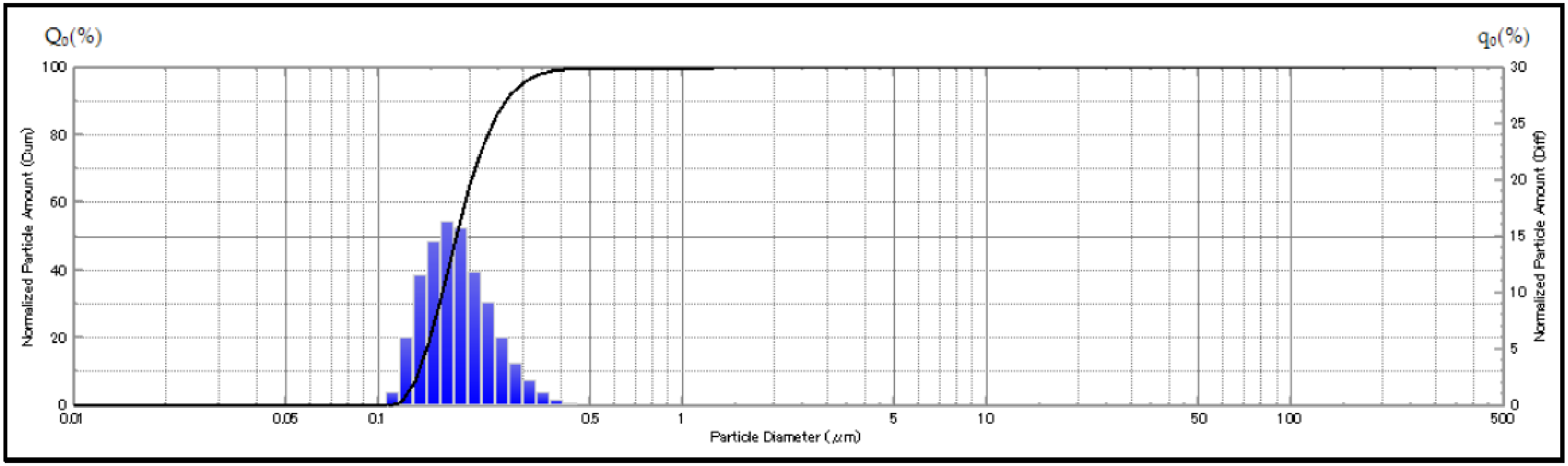

C

File Name	After ultrasonication – 9 min	Meas Date	18/05/12 18:54:06
Sample ID		Sample No.	
Comment			

Median D	177.317	Mean V	179.318
Modal D	177.828	Std Dev	0.107

10%D(μm)	50%D(μm)	90%D(μm)	0%D(μm)	0%D(μm)	0%D(μm)	0%D(μm)	0%D(μm)	0%D(μm)
130.533	177.317	251.167	0.000	0.000	0.000	0.000	0.000	0.000

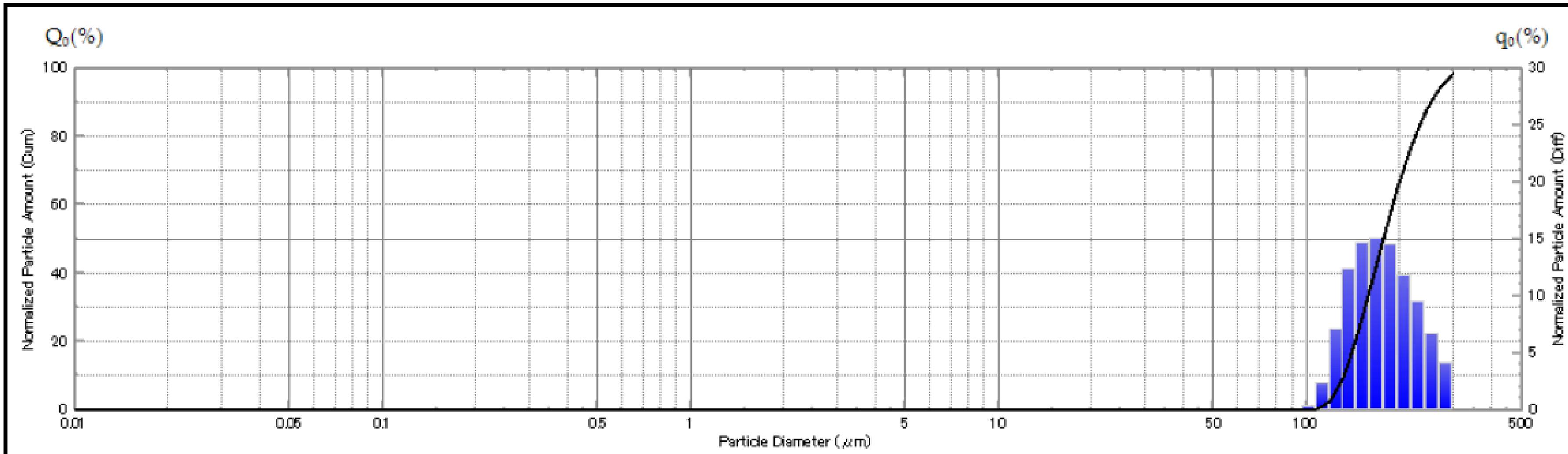

D

File Name	Before ultrasonication – 9 min	Meas Date	18/05/12 19:00:58
Sample ID		Sample No.	
Comment			

Median D	218.656	Mean V	217.831
Modal D	223.872	Std Dev	0.078

10%D(μm)	50%D(μm)	90%D(μm)	0%D(μm)	0%D(μm)	0%D(μm)	0%D(μm)	0%D(μm)	0%D(μm)
167.880	218.656	285.893	0.000	0.000	0.000	0.000	0.000	0.000

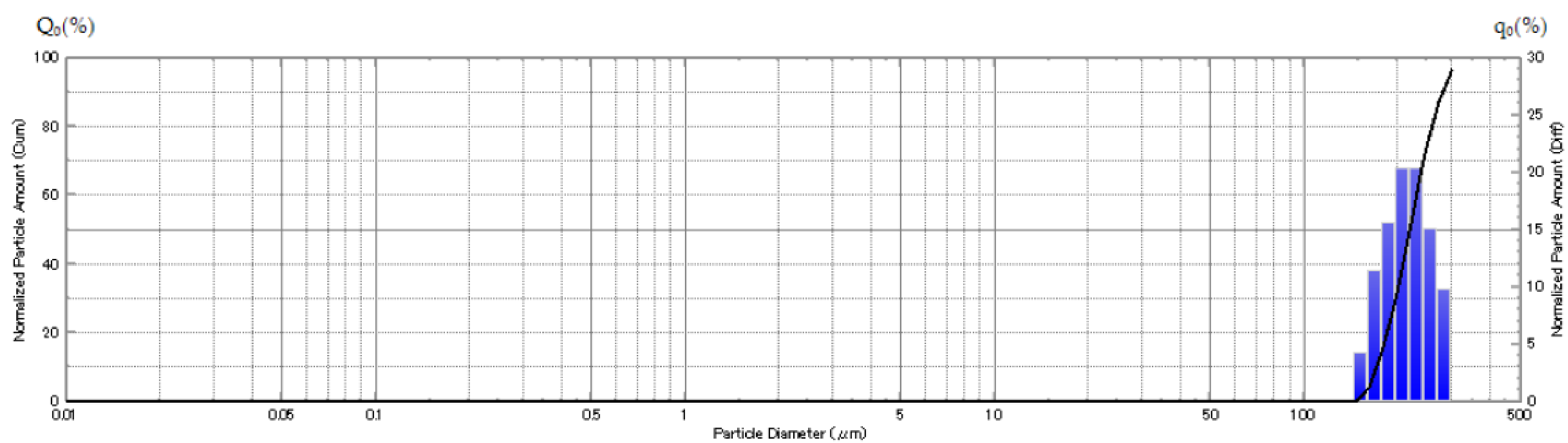

E

File Name	During ultrasonication – 9 min	Meas Date	18/05/12 19:18:42
Sample ID		Sample No.	
Comment			

Median D	0.151	Mean V	0.155
Modal D	0.141	Std Dev	0.114

10%D(μm)	50%D(μm)	90%D(μm)	0%D(μm)	0%D(μm)	0%D(μm)	0%D(μm)	0%D(μm)	0%D(μm)
0.112	0.151	0.213	0.000	0.000	0.000	0.000	0.000	0.000

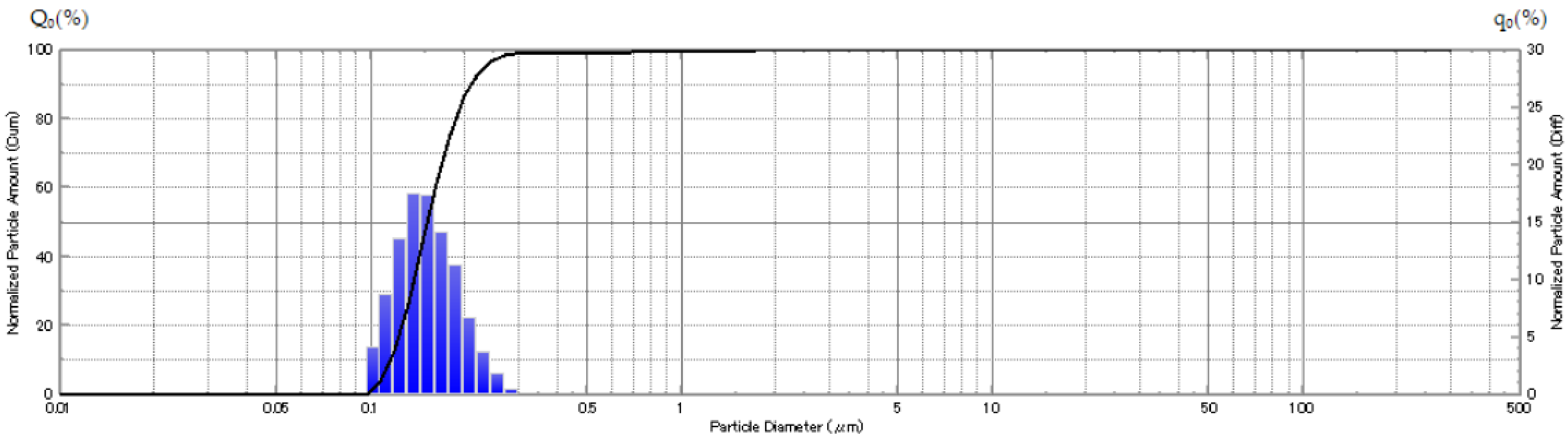

F

File Name	After ultrasonication – 9 min	Meas Date	18/05/12 19:28:24
Sample ID		Sample No.	
Comment			

Median D	0.152	Mean V	0.156
Modal D	0.141	Std Dev	0.114

10%D(μm)	50%D(μm)	90%D(μm)	0%D(μm)	0%D(μm)	0%D(μm)	0%D(μm)	0%D(μm)	0%D(μm)
0.113	0.152	0.214	0.000	0.000	0.000	0.000	0.000	0.000

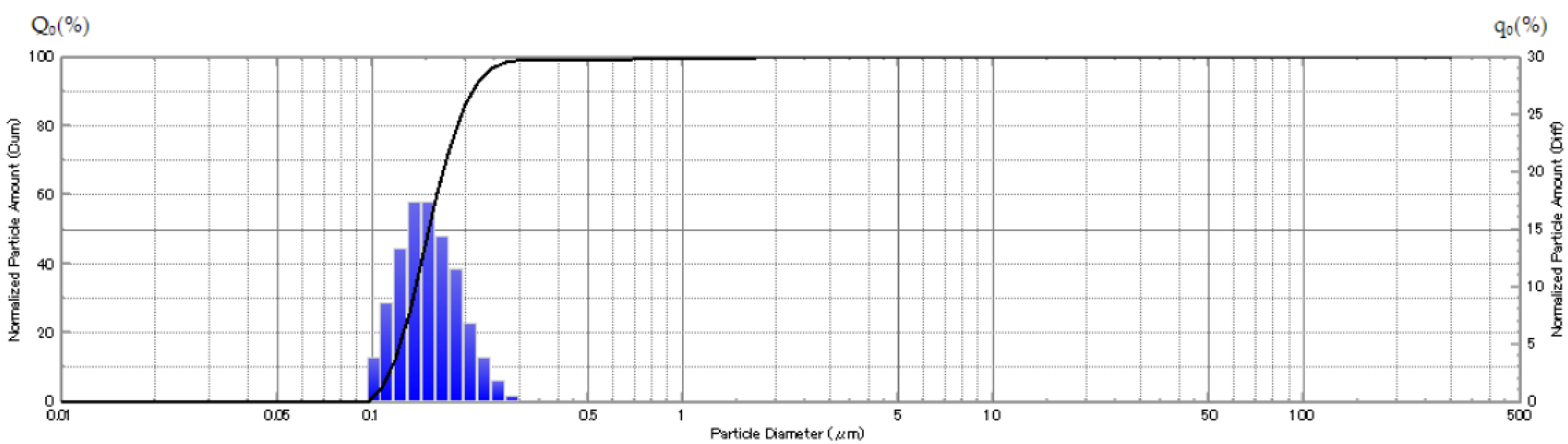

G

File Name	Before ultrasonication – 9 min	Meas Date	18/05/14 13:47:33
Sample ID		Sample No.	
Comment			

Median D	81.073	Mean V	82.083
Modal D	70.795	Std Dev	0.112

10%D(μm)	50%D(μm)	90%D(μm)	0%D(μm)	0%D(μm)	0%D(μm)	0%D(μm)	0%D(μm)	0%D(μm)
57.900	81.073	117.937	0.000	0.000	0.000	0.000	0.000	0.000

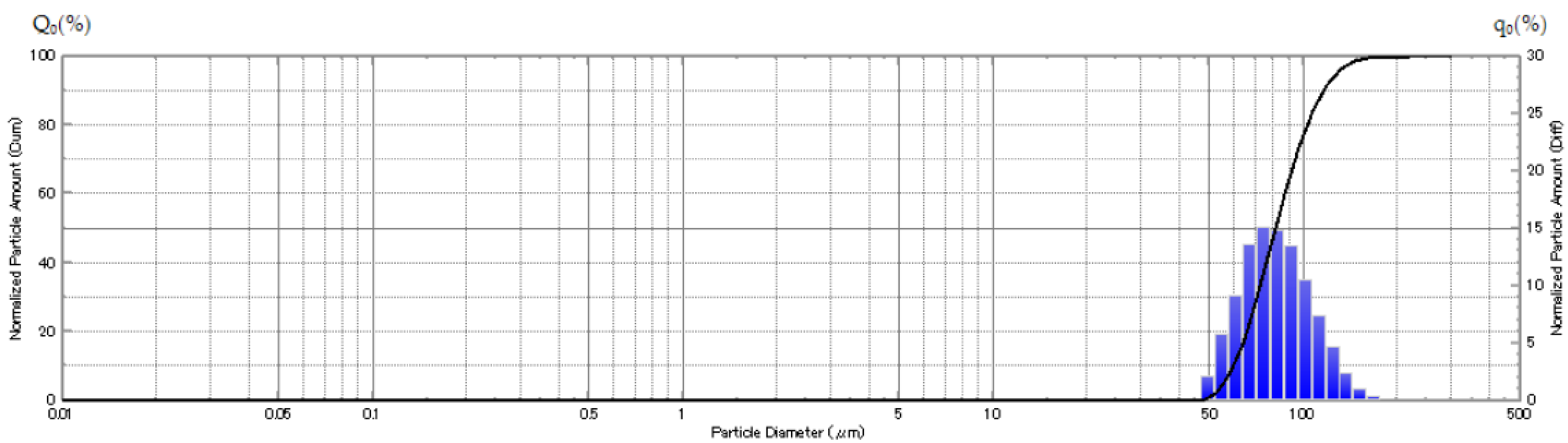

H

File Name	During ultrasonication – 9 min	Meas Date	18/05/14 13:57:24
Sample ID		Sample No.	
Comment			

Median D	0.015	Mean V	0.016
Modal D	0.011	Std Dev	0.132

10%D(μm)	50%D(μm)	90%D(μm)	0%D(μm)	0%D(μm)	0%D(μm)	0%D(μm)	0%D(μm)	0%D(μm)
0.011	0.015	0.024	0.000	0.000	0.000	0.000	0.000	0.000

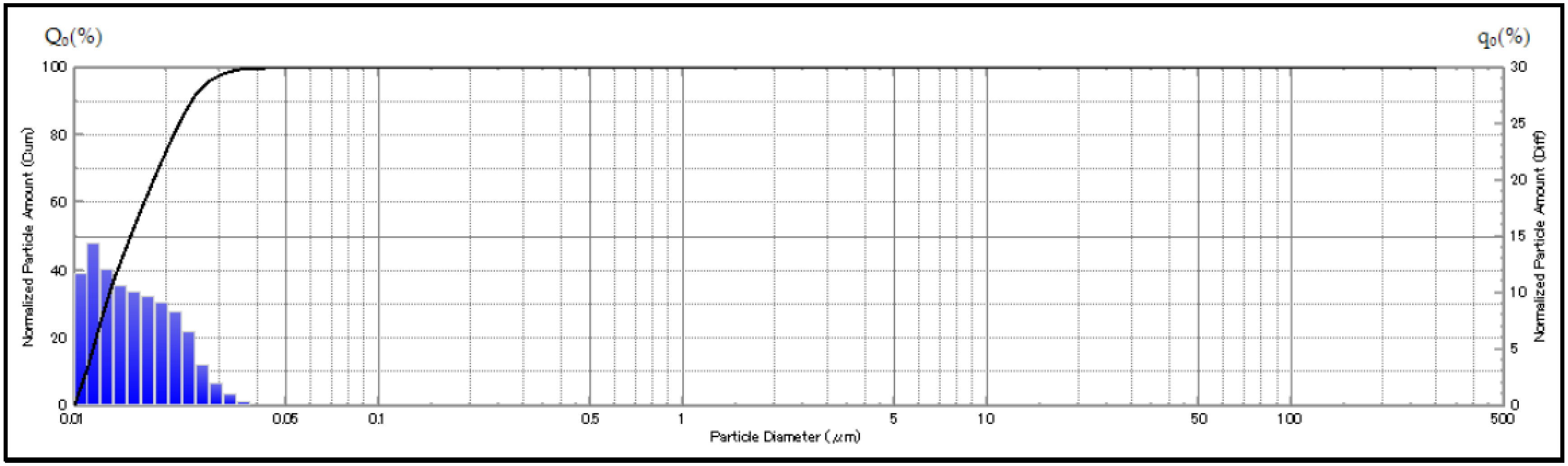

I

File Name	After ultrasonication – 9 min	Meas Date	18/05/14 14:07:01
Sample ID		Sample No.	
Comment			

Median D	83.023	Mean V	83.755
Modal D	89.125	Std Dev	0.110

10%D(μm)	50%D(μm)	90%D(μm)	0%D(μm)	0%D(μm)	0%D(μm)	0%D(μm)	0%D(μm)	0%D(μm)
59.629	83.023	119.299	0.000	0.000	0.000	0.000	0.000	0.000

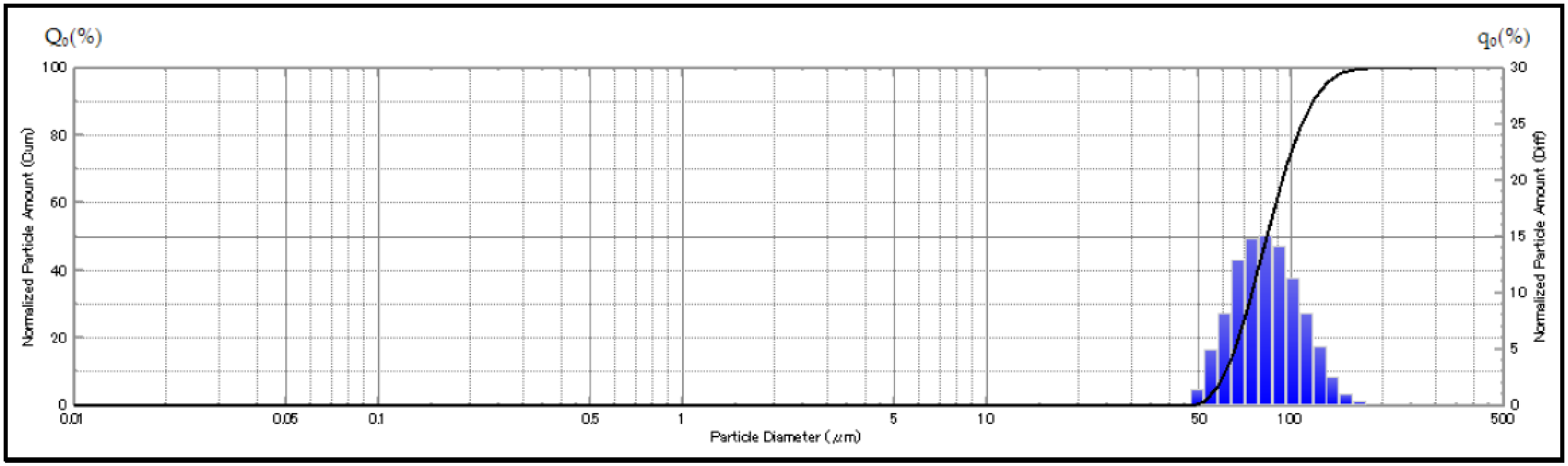

Supplementary Figure 1. Laser diffraction characterization of TiO_2 particle agglomeration and dispersion. Representative volume-weighted (Q_3) particle-size distributions obtained by SALD-7101 laser diffraction analysis of three independent TiO_2 preparations. Panels A–C, D–F, and G–I correspond to preparations 1, 2, and 3, respectively, and show representative measurements obtained under continuous circulation without ultrasonication, during ultrasonication (40 kHz, 40 W), and after termination of ultrasonication. Complete particle-size measurements across the 1–9 min measurement period are provided in Supplementary Table S1. The large particle-size values recorded for preparation 3 in the absence of ultrasonication were noted in the original experimental record as potentially affected by air-bubble formation.

Supplementary Table S1. Particle-size measurements of three independent TiO_2 preparations

Measurement	TiO_2 Preparation #1		TiO_2 Preparation #2		TiO_2 Preparation #2	
	D50 (µm)	Dmean (µm)	D50 (µm)	Dmean (µm)	D50 (µm)	Dmean (µm)
Without ultrasonication – 1 min	0.192	0.215	218.656	217.831	86.092	87.149
Without ultrasonication – 3 min	0.187	0.209	218.656	217.831	84.127	85.199
Without ultrasonication – 5 min	195.664	195.818	218.656	217.831	81.523	82.632
Without ultrasonication – 7 min	200.560	200.146	218.656	217.831	82.899	83.635
Without ultrasonication – 9 min	192.965	193.404	218.176	217.378	81.073	82.083
During ultrasonication – 1 min	0.215	0.228	0.184	0.189	0.222	0.236
During ultrasonication – 3 min	0.206	0.214	0.161	0.165	0.188	0.195
During ultrasonication – 5 min	0.2	0.207	0.157	0.162	0.017	0.018
During ultrasonication – 7 min	0.191	0.197	0.156	0.161	0.015	0.016
During ultrasonication – 9 min	0.18	0.185	0.151	0.155	0.015	0.016
After ultrasonication – 1 min	0.179	0.183	0.151	0.155	83.750	84.494
After ultrasonication – 3 min	171.621	174.311	0.151	0.155	84.003	84.975
After ultrasonication – 5 min	169.247	172.263	0.151	0.155	81.680	82.125
After ultrasonication – 7 min	170.846	173.640	0.152	0.156	81.679	82.622
After ultrasonication – 9 min	177.317	179.318	0.152	0.156	83.023	83.755
