# Supplementary material for: TiO2 particles induce CHIT1 and YKL-40 in human macrophages: implications for implant-associated inflammation
Source: Front Immunol. 2026 Oct 28;17:1964504. doi: 10.3389/fimmu.2026.1964504 (PMC13751356; doi:10.3389/fimmu.2026.1964504)
Supplement: Supplementary file 2 [file DataSheet2.pdf]

Supplementary Table S2. Clinical characteristics of peri-implant tissue specimens

**The anatomical location and indication recorded for this specimen appear inconsistent and should be verified against the original clinical record before publication.*

Patient number	Implant type	Anatomical location	Indication for implantation	Documented complication
1	Titanium plate	Left clavicle	Clavicle fracture	None reported.
2	Titanium clavicular hook plate	Clavicle	Clavicle fracture	Persistent pain
3	Titanium plate	Right clavicle	Clavicle fracture	None reported.
4	Titanium locking compression plate (LCP)	Proximal tibia	Tibial fracture	Mechanical irritation.
5	Titanium plate	Distal tibial shaft	Tibial fracture	None reported.
6	Titanium plate	Right lateral clavicle*	Tibial fracture	None reported.
7	Titanium plate	Upper ankle joint	Ankle fracture	None reported.
8	Titanium plate	Ankle/fibula	Ankle fracture	None reported.
